# Wearable Artificial Intelligence for Epilepsy: Scoping Review

**DOI:** 10.2196/73593

**Published:** 2025-10-31

**Authors:** Sarah Aziz, Amal A M Ali, Hania Aslam, Noor ul Ain, Amna Tariq, Zain Sohail, Sofia Murtaza, Hafiza Iqra Mahmood, Muhammad Irfan Wazeer, Fozia Murtaza, Alaa Abd-alrazaq, Mohammed Alsahli, Rafat Damseh, Rawan AlSaad, Tariq Shahzad, Arfan Ahmed, Javaid Sheikh

**Affiliations:** 1 AI Center for Precision Health, Weill Cornell Medical College in Qatar, Education City, Street 2700, Doha, 24144, Qatar, 974 4492 8827; 2 Social and Economic Survey Research Institute, Qatar University, Doha, Qatar; 3 Department of Computer Science, COMSATS University Islamabad, Sahiwal, Pakistan; 4 Department of Home Economics, University of Agriculture, Faisalabad, Pakistan; 5 Health Informatics Department, College of Health Sciences, Saudi Electronic University, Riyadh, Saudi Arabia; 6 Department of Computer Science and Software Engineering, United Arab Emirates University, Abu Dhabi, United Arab Emirates; 7 Department of Computer Engineering, COMSATS University Islamabad, Sahiwal, Pakistan

**Keywords:** wearable AI, epilepsy, seizure, machine learning, artificial intelligence, wearable devices, scoping review

## Abstract

**Background:**

Epilepsy affects approximately 50 million people globally and imposes a substantial clinical and societal burden, requiring continuous and personalized monitoring for effective management. Wearable artificial intelligence (AI) technologies offer a promising solution by leveraging physiological signals and machine learning for seizure detection and prediction. While various approaches have been proposed, a comprehensive overview summarizing these advances and challenges is still needed.

**Objective:**

This review aims to comprehensively explore and map the existing literature on AI-driven wearable technologies for epilepsy, identifying device characteristics, AI methodologies, biosignal measurements, validation approaches, and research gaps.

**Methods:**

A scoping review was conducted following the PRISMA-ScR (Preferred Reporting Items for Systematic Reviews and Meta-Analyses Extension for Scoping Reviews) guidelines. A systematic search was performed across six electronic databases (Scopus, MEDLINE, Embase, ACM Digital Library, IEEE Xplore, and Google Scholar) to identify relevant studies published up to December 2023. We included studies that developed AI algorithms for epilepsy using noninvasive wearable devices (eg, smartwatches, smart clothing) and excluded those using nonwearables or in-body devices. Eligible publication types included journal articles, conference papers, and dissertations. Study selection and data extraction were performed independently by six reviewers. The extracted data were synthesized narratively.

**Results:**

A total of 67 studies met the inclusion criteria. Research in this domain has increased significantly since 2021, with India, the United States, and China leading contributions. The studies examined both commercial (n=31, 46.3%) and noncommercial (n=31, 46.3%) wearable devices, with Empatica smart bands being the most frequently used. The primary biosignals monitored included activity measures (n=36, 53.7%), cardiovascular metrics (n=33, 49.3%), brain activity (n=24, 35.8%), and skin conductance (n=23, 34.3%). The most common AI models were support vector machines (n=28, 41.8%), random forests (n=14, 20.9%), and convolutional neural networks (n=10, 14.9%). Most models focused on seizure detection (n=54, 80.6%) compared to seizure prediction (n=14, 20.9%), reflecting a research imbalance that suggests the need for further development in predictive analytics. Sensitivity (n=54, 80.6%) was the most frequently reported performance metric, indicating a focus on identifying seizures; however, comprehensive clinical validation remains limited. Closed-source data predominated (n=44, 65.7%), limiting the generalizability of findings. The most used validation methods were leave-one-out cross-validation (n=21, 31.3%) and k-fold cross-validation (n=20, 29.9%), while video electroencephalography served as the primary reference standard (n=35, 52.2%).

**Conclusions:**

Wearable AI technologies show significant promise in epilepsy management, offering real-time, continuous monitoring and early seizure detection. To realize clinical impact, future research should prioritize the standardization of validation methods, promote open data exchange for reproducibility, and develop energy-efficient algorithms that support real-world deployment in wearable devices.

## Introduction

### Background

Epilepsy affects an estimated 50 million individuals worldwide and remains one of the most common serious neurological disorders, often leading to significant physical injury, psychological distress, and reduced quality of life [1]. The condition’s prevalence exhibits geographical variation, with a higher incidence in low-income nations, often attributed to increased risk factors such as neurocysticercosis, perinatal complications, and limited health care access. Epilepsy encompasses various seizure types categorized broadly as focal, generalized, combined focal and generalized, or unknown onset epilepsy, each affecting patients differently [2].

Accurate diagnosis and prompt management tailored to specific seizure types are crucial to minimizing risks and improving patient-related outcomes. Precise detection enables health care providers to develop tailored treatment strategies, potentially incorporating pharmacological interventions, lifestyle modifications, and, in certain cases, surgical procedures to achieve effective seizure control [3]. The current seizure detection techniques primarily focus on clinical assessments and electroencephalography (EEG). The former relies on patient histories and observed seizure events, while EEG records cerebral electrical activity to identify epilepsy-associated abnormalities. However, these methods have inherent limitations, such as limited accessibility, particularly in resource-constrained environments. EEG procedures are often time-intensive and expensive, and require patients to visit specialized centers. Moreover, the absence of seizure activity during EEG recording can yield inconclusive outcomes, underscoring the need for accessible and continuous monitoring solutions [4]. Given these limitations of conventional EEG, artificial intelligence (AI) emerges as particularly suitable for seizure detection due to its ability to analyze complex physiological data continuously and in real time. AI methods, such as machine learning algorithms, can automatically recognize subtle patterns within large volumes of physiological signals, enabling timely and accurate seizure detection and prediction. When integrated into wearable devices, AI offers a highly accessible and user-friendly approach that addresses the challenges of traditional EEG-based methods [4].

Wearable AI-based devices represent a promising advancement in addressing the limitations of traditional epilepsy monitoring. These smart devices—such as smartwatches, wristbands, smart textiles, and head-mounted sensors—are designed to be worn on or near the body and enable continuous, noninvasive tracking of physiological data [5]. By leveraging real-time data collection and embedded AI algorithms, they can detect subtle changes in biosignals (eg, heart rate, movement, skin conductance) that may help to indicate the onset of a seizure. This can also facilitate early warnings and timely medical interventions, especially in unsupervised or at-home settings. Compared to conventional EEG, wearable AI solutions’ integration into daily life holds potential benefits to enhance patient autonomy, safety, and overall quality of life [5].

This scoping review aims to map the current landscape of noninvasive, AI-driven wearable technologies for epilepsy detection and prediction, thereby providing a structured foundation for future investigations and facilitating translation of promising solutions into clinical practice.

### Research Problem and Aim

Despite increasing interest in AI-based wearables for epilepsy, existing reviews remain fragmented and limited in scope. While several literature reviews have been conducted on this topic, they have been characterized by certain limitations. First, none of these reviews specifically targeted the intersection of AI and epilepsy detection [6-11]. Second, the reviews did not encompass data collected through wearable devices [7,8,10,12,13]. Third, a significant portion of previous studies narrowly focused on a particular data type, predominantly EEG [7,12,13]. Fourth, the majority of these studies were conventional literature reviews rather than more comprehensive scoping or systematic reviews [7,9,10,12,13]. Lastly, the search strategies employed in these reviews often overlooked key databases such as CINAHL, PsycINFO, Scopus, IEEE, and the ACM digital library [6,8,10,11].

To address these gaps, the present review offers the first scoping review that systematically maps AI-driven, noninvasive wearable technologies used for epileptic seizure detection and prediction. The major contributions of this work include (1) mapping the landscape of wearable device types, biosignal modalities, and AI methodologies; (2) identifying validation frameworks and performance metrics; (3) identifying current research limitations and gaps; and (4) providing future directions, including clinical translation, regulatory challenges, and ethical considerations. This review aims to serve as a comprehensive and foundational resource to inform future research, development, and implementation of wearable AI systems in epilepsy management.

## Methods

To achieve the objectives of this study, a scoping review was conducted following the guidelines of the PRISMA-ScR (Preferred Reporting Items for Systematic Reviews and Meta-Analyses Extension for Scoping Reviews) [14]; the PRISMA-ScR checklist associated with this review can be found in Checklist 1. The following sections provide a detailed description of the methods used in this review.

### Search Strategy

To identify relevant studies, we performed searches across six electronic databases on December 7, 2023: Scopus, MEDLINE (via Ovid), Embase (via Ovid), ACM Digital Library, IEEE Xplore, and Google Scholar. These databases were selected to cover both clinical/biomedical (MEDLINE, Embase, and Scopus) and technical/engineering (ACM and IEEE) literature, ensuring broad coverage across disciplines relevant to AI and wearable technologies. We also set up a monthly automated search to run for 6 months, concluding on June 7, 2024. Given the large number of results generated by Google Scholar, which sorts entries by relevance, we limited our review to the top 100 entries (10 pages) to capture the most pertinent literature while maintaining feasibility and avoiding redundancy among different database searches. To ensure comprehensive coverage, we also performed backward and forward reference checking of included articles. Our search query consisted of three primary categories of terms: terms related to AI, terms related to epilepsy, and terms related to wearable devices. Our search query consisted of three key categories: terms related to AI, epilepsy, and wearable devices. Full search strategies for each database are included in Multimedia Appendix 1.

### Study Eligibility Criteria

This review considered studies that developed AI algorithms for epilepsy using data from noninvasive, body-worn wearable devices (eg, smartwatches, smart glasses, smart clothing, smart bracelets, smart tattoos). We excluded studies using nonwearables, handheld devices (eg, smartphones used independently), near-body wearables (eg, devices placed near but not on the body), and in-body wearables (eg, implants). Wired wearable systems were also excluded. We specifically included all AI algorithms used for any aspect of epilepsy, including diagnosis, monitoring, screening, therapy, prediction, and prevention. We included studies that combined data from wearable devices with other sources (eg, nonwearable devices, questionnaires, and interviews). We excluded studies that only presented a theoretical framework for AI-based wearable devices for epilepsy. Eligible publication types were peer-reviewed journal articles, conference papers, and dissertations written in English. We excluded reviews, preprints, conference abstracts, posters, protocols, editorials, and commentaries. No restrictions were imposed on measured outcomes, settings, publication dates, or countries of publication.

### Study Selection Process

The study selection process was carried out in three stages. First, duplicates were removed from the retrieved studies using EndNote X9, followed by a manual duplicate check to ensure accuracy. Next, six reviewers independently screened titles and abstracts for relevance. Finally, the same six reviewers independently evaluated the full texts using predefined eligibility thresholds: the study had to involve AI methods, epilepsy-focused objectives, and data derived from wearable devices as per the criteria above. Discrepancies were resolved by a seventh reviewer.

### Data Extraction Process

Six reviewers independently used Microsoft Excel to extract data on study metadata, wearable devices, epilepsy, and AI techniques. Any discrepancies between the reviewers were resolved through another reviewer by discussion. The extraction template included fields for study design, population, device characteristics, signal modalities, AI models, performance metrics, and clinical applications. The data extraction form can be found in Multimedia Appendix 2.

### Data Synthesis

Data extracted from the included studies were synthesized using a narrative approach, where information was summarized and presented through text, tables, and figures. To structure the synthesis, studies were categorized according to (1) the type of wearable device used (eg, wrist-worn, head-mounted), (2) biosignal modality (eg, EEG, photoplethysmography, electrodermal activity, accelerometer), (3) AI technique (eg, machine learning, deep learning), and (4) application context (eg, seizure detection, prediction, monitoring). The study began by outlining the metadata of the studies (eg, year and country of publication). Next, the characteristics of wearable devices used in the studies were outlined, including their status, type, placement, and operating system. Finally, the features of the AI techniques applied were detailed in terms of algorithms used, their objectives, dataset size, data input type, and model performance. Patterns and trends across categories were identified inductively through iterative comparison of extracted data.

Conflicting findings between studies (eg, differences in model accuracy or signal reliability) were documented and interpreted based on study context, population, data quality, and validation method. These contradictions were discussed in the Results and further reflected upon in the Discussion and Limitations sections. Data synthesis was managed using Microsoft Excel.

## Results

### Search Results

**Figure 1.**
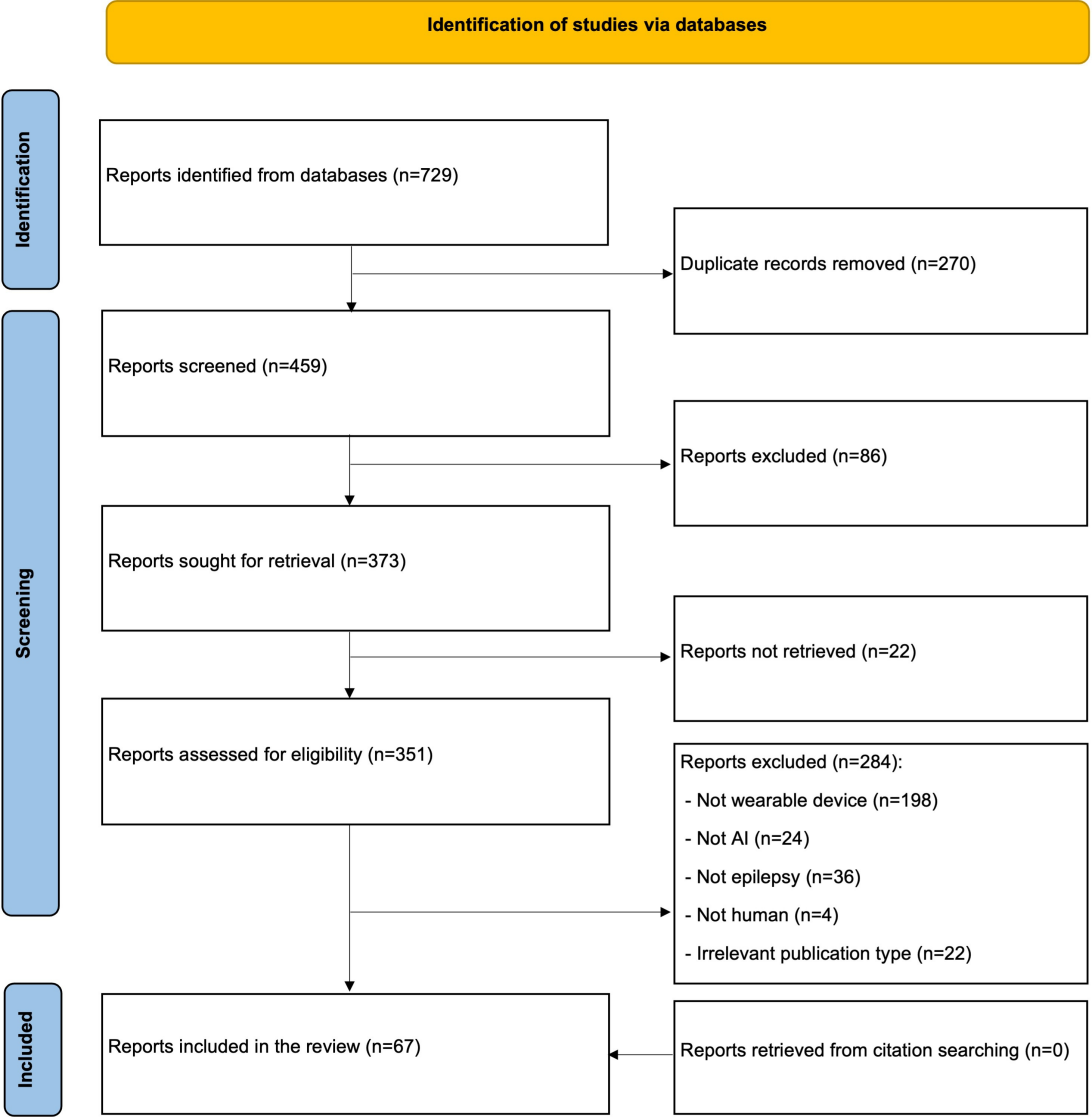
Flowchart of the study selection process.

Figure 1 shows that when the above-mentioned databases were searched, 729 citations were returned. A total of 270 duplicates were identified and deleted using EndNote X9, leaving 459 studies. In addition, 87 studies were excluded after screening the titles and abstracts of these 459 studies. About 22 full-text studies were not able to be retrieved. After retrieving and reviewing the entire text of all 351 remaining studies, it was decided that 284 of them were ineligible for inclusion. The primary grounds for elimination were that they did not use wearable devices (n=198, 69.7%) or AI algorithms (n=24, 8.5%), were of unrelated publication genres (n=22, 7.7%), were not epilepsy-related research (n=36, 12.7%), or were nonhuman research (n=4, 1.4%). The study found no new studies related to this review by examining the reference list of the included studies. This review included a total of 67 studies [15-81].

### Characteristics of Included Studies

Table 1 provides an overview of the characteristics of the 67 included studies. Studies included in this analysis were published between 2013 and 2024. A high proportion of the articles were published in 2023 (n=16, 23.9%), followed by 2022 (n=14, 20.9%) and 2021 (n=13, 19.4%). In particular, 2016 and the early years have the smallest percentage of publications (n=4, 6.0%), which indicates an increasing interest in research in recent years. Geographically, India accounted for the largest number of studies (n=12, 17.9%), followed by the United States (n=9, 13.4%), China (n=8, 11.9%), Switzerland, Germany, Belgium, and Australia (each at n=5, 7.5%). According to the type of publication, most of the included studies were published as journal articles (n=40, 59.7%), while the remaining were conference papers (n=27, 40.3%). Multimedia Appendix 3 provides the characteristics of each included study.

**Table 1.** Characteristics of the included studies (n=67 studies).

Features	Studies, n (%)	References
Year of publication		
2024	1 (1.5)	[36]
2023	16 (23.9)	[18,19,27,37,39,46,50,65-67,71,72,74-76,79]
2022	14 (20.9)	[15,21,24,28,31,35,42,44,48,51,61,69,77,80]
2021	13 (19.4)	[22,25,26,29,34,41,43,60,62,64,70,73,78]
2020	3 (4.5)	[47,56,59]
2019	7 (10.4)	[23,33,49,53,55,68,81]
2018	6 (9.0)	[17,30,32,40,52,54]
2017	3 (4.5)	[16,38,63]
≤2016^a^	4 (6.0)	[20,45,57,58]
Country of publication		
India	12 (17.9)	[15,27,35,41,42,47,55,64,66,67,69,74]
United States	9 (13.4)	[17,18,39,43,59,62,75,76,81]
China	8 (11.9)	[28,36,40,48,65,72,77,78]
Switzerland	5 (7.5)	[19,24,32-34]
Germany	5 (7.5)	[21-23,45,56]
Belgium	5 (7.5)	[38,57,58,73,80]
Australia	5 (7.5)	[25,53,54,70,79]
Italy	2 (3.0)	[63,68]
Sweden	2 (3.0)	[46,49]
United Kingdom	2 (3.0)	[51,52]
Other (n=1)^b^	12 (17.9)	[16,20,26,29-31,37,44,50,60,61,71]
Publication type		
Conference paper	27 (40.3)	[15-17,23,27,30-32,34,39-42,45,46,50,54,55,58,60,66,67,69,74,77,78,81]
Journal article	40 (59.7)	[18-22,24-26,28,29,33,35-38,43,44,47-49,51-53,56,57,59,61-65,68,70-73,75,76,79,80]

a Includes studies published in 2016 or earlier that add up to one study.

b Includes studies from several countries that add up to one study.

### Participant Demographics Across Included Studies

Table 2 shows the demographic characteristics of the participants who were included in the study. The results indicate that 85.1% (n=57) of the articles reported the number of participants, which ranged from 1 to 400, with an average of 57 (SD 75.5) participants. The high SD value reflects a wide variation in sample sizes. Studies that did not report the number of participants were excluded from the average calculation (n=10, 14.9%). Regarding gender distribution, 40.3% (n=27) of the articles reported that the proportion of female participants ranged from 22% to 77%, with an average of 49.1% (SD 13.8%). However, 59.7% (n=40) of the studies did not provide the gender information, which presents a significant limitation in assessing the demographic balance. Additionally, only 26.9% (n=18) of the articles reported the age of the participants, with an average age of 25.4 (SD 12.7) years. The age range across studies extended from 1 month to 80 years, based on the lowest and highest age values reported. However, some of the articles did not report the participants’ exact age but mentioned participant age groups. The results of these articles showed that the age group of adult participants is most frequently included (n=31, 46.3%) because this category covers a wide age range from 19 to 64 years, followed by children (n=24, 35.8%) and older people (n=7, 10.4%), while 38.8% (n=26) do not specify the age groups of participants. Multimedia Appendix 3 provides the characteristics of each included study.

**Table 2.** Demographic characteristics of participants (n=67 studies).

Features	Studies, n (%)	Mean (SD)^a^	Range	References
Number of participants				
Reported	57 (85.1)	57.0 (75.5)	1‐400	[15-26,28-39,43-45,48,49,51,53-68,70-77,79-81]
Not reported	10 (14.9)	—^b^	—	[27,40-42,46,47,50,52,69,78]
Age (y)				
Reported	18 (26.9)	25.4 (12.7)	5.7‐45	[21,22,24,25,29,36-38,43,48,54-56,59,62,71,76,77]
Not reported	49 (73.1)	—	—	[15-20,23,26-29,31-35,39-42,44-47,49-53,57,58,60,61,63-70,72-75,78,79,81]
Female participants				
Reported	27 (40.3)	49.1 (13.8)	22‐77	[19,21,22,24,25,28,29,35-38,43,44,49,51,54-56,59,61-63,71,75-77,79]
Not reported	40 (59.7)	—	—	[15-18,20,23,26,27,30-34,39-42,45-48,50,52,53,57,58,60,64,66-70,72-74,78,80,81]
Participants age groups				
Children	24 (35.8)	—	0‐18	[21,22,24,25,28,29,36,44,48,55-59,63-65,68,72,75-77,79]
Adult	31 (46.3)	—	19‐64	[20-22,25,26,28,29,32-34,37,38,43,44,49,51,53-56,59,61-65,68,70,72,77]
Elderly	7 (10.4)	—	≥65	[19-22,44,49,61]
Not reported	26 (38.8)	—	—	[15-18,23,30,31,35,39-42,45-47,50,52,60,66,67,69,73,74,78,80,81]

a All means were calculated based on studies that reported the relevant data.

b Not applicable.

### Characteristics of Wearable Devices

Table 3 shows the characteristics of wearable devices, including their commercial status, device types, and placement. The result shows that the commercial (n=31, 46.3%) and noncommercial (n=31, 46.3%) wearable devices have equal percentages, with 7.5% (n=5) of studies not reporting the state of the device. Among the wearable device names, Empatica was the most commonly used (n=15, 22.4%), followed by Byteflies Kit (n=5, 7.5%), and 43.3% (n=29) did not report device names. Smart bands (n=24, 35.8%) were the most common type of wearable devices, followed by wearable sensors (n=18, 26.9%) and EEG devices (n=12, 17.9%). Regarding the placement of the wearable device, the result showed that half of the studies reported the devices being placed on the wrists (n=34, 50.7%), followed by the head (n=15, 22.4%), in the ear (n=10, 14.9%), and on the ankle (n=8, 11.9%). The features of wearable devices in each included study are presented in Multimedia Appendix 4.

**Table 3.** Characteristics of the wearable devices (n=67 studies).

Features	Studies, n (%)	References
Status of wearable device^a^		
Commercial	31 (46.3)	[15,17,18,20-22,31,35,39,41,45,46,48-50,55,56,59,61-63,66,68,70,71,73,75,76,79-81]
Noncommercial	31 (46.3)	[16,19,23-25,27-30,32-34,37,38,40,42-44,47,51-54,57,58,60,64,65,67,77,78]
Not reported	5 (7.5)	[26,36,69,72,74]
Name of wearable device		
Empatica	15 (22.4)	[17,21,22,31,35,39,45,56,62,63,68,75,76,79,81]
Byteflies Kit	5 (7.5)	[18,31,46,73,80]
Fitbit	2 (3.0)	[59,70]
SmartCardia INYU	2 (3.0)	[32,33]
Other (n=1)^a^	21 (31.3)	[15,19,20,27,31,34,36,37,48-50,61,63-66,68,71]
Not reported	29 (43.3)	[16,23-26,28-30,38,40-44,47,51,53-55,57,58,60,67,69,72,74,77,78]
Type of wearable device		
Smart band	24 (35.8)	[15-17,28,31,45,48,49,53,54,56,57,60,63,64,68,71,75-79,81]
Wearable sensor	18 (26.9)	[18,20,27,31,37,43,44,46,51,55,56,58,67,69,72-74,80]
EEG^b^ device^c^	12 (17.9)	[23-26,29,32-34,38,40-42]
Smartwatch	7 (10.4)	[21,22,35,39,47,59,70]
Other (n<3)^d^	6 (9.0)	[18,19,30,50,61,65]
Not reported	3 (4.5)	[36,52,66]
Placement of the wearable device		
Wrist	34 (50.7)	[15-17,21,22,28,31,35,39,42-45,47,49,53,54,56-60,62,63,68-70,72,75-79,81]
Head	15 (22.4)	[19,23-26,29,31,37,41,55,64,65,71,72,74]
Ear	10 (14.9)	[18,27,38,43,44,61,71,73,74,80]
Ankle	8 (11.9)	[42,48,56-58,69,75,76]
Chest	6 (9.0)	[16,33,34,43,44,72]
Arm	5 (7.5)	[20,30,43,44,63]
Back	2 (3.0)	[18,80]
Other	8 (11.9)	[20,50,51,61,72]
Not reported	7 (10.4)	[32,36,40,46,52,66,67]

a Include different names of the wearable devices that add up to one study.

b EEG: electroencephalogram.

c EEG devices refer to EEG-based wearable technologies.

d Include different types of wearable devices that add up to less than three studies.

Table 4 shows the types of biosignals measured, sensors used, and sensing methods applied in the 67 included studies on wearable devices. The wearable devices of the included studies measured various biosignals. The most commonly measured biosignal was activity measurement (n=36, 53.7%), followed by cardiovascular measurement (n=33, 49.3%), brain activity (n=24, 35.8%), and skin conductance (n=23, 34.3%). On the other hand, the less commonly used biosignals include acoustic (n=3, 4.5%), orientation (n=3, 4.5%), respiratory measures (n=3, 4.5%), and sleep measures (n=1, 1.5%), which were less common. The wearable devices used various types of sensors, with accelerometers being the most frequent (n=41, 61.2%), followed by electroencephalogram sensors (n=24, 35.8%), electrodermal sensors (n=21, 31.3%), and photoplethysmography (n=18, 26.9%). The majority of studies (n=63, 94.0%) relied on an opportunistic approach for data sensing, with data automatically collected by devices without active user input and often collected through sensors embedded in mobile phones and wearables. A small number of studies (n=1, 1.5%) used a participatory approach, in which users actively contributed data by means of manual input, self-reporting, or signal reaction. The remaining 4.5% (n=3) of the studies did not clearly indicate the types of sensing methods used. The sensors of wearable devices included in each study are presented in Multimedia Appendix 4.

**Table 4.** Features of the wearable devices (n=67 studies).

Features	Studies, n (%)	References
Measured biosignal^a^		
Activity measures	36 (53.7)	[15,20-22,27,28,30,31,35,36,39,42-45,47-50,53-58,60-63,67-70,76,77,81]
Cardiovascular measures	33 (49.3)	[16-19,21,22,27,31-35,39,42-44,48,50,55,56,59,61,62,67-70,72-75,78,80]
Brain activity	24 (35.8)	[18,19,23-26,29,31,37,38,40-42,46,52,55,61,64,65,69,71-73,79]
Skin conductance	23 (34.3)	[16,17,21,22,27,31,35,36,39,42,43,45,56,62,63,67-69,74-76,78,80]
Skin temperature	13 (19.4)	[17,27,31,35,39,44,56,62,68,74,76,78,80]
Neuromuscular activity	8 (11.9)	[30,31,36,43,44,57,72,76]
Acoustic	3 (4.5)	[27,43,51]
Orientation	3 (4.5)	[28,36,76]
Respiratory measures	3 (4.5)	[16,27,50]
Sleep measures	1 (1.5)	[70]
Not reported	1 (1.5)	[66]
Sensors		
Accelerometer	41 (61.2)	[15,16,20-22,28,30,31,35,36,39,43-45,47-51,53-62,66-68,70-73,76-79,81]
Electroencephalogram	24 (35.8)	[18,19,23-26,29,37,38,40-42,46,52,55,61,64,65,69,71-74,80]
Electrodermal	21 (31.3)	[16,17,21,22,27,31,35,36,39,43,45,48,56,62,63,67-69,75-77,79,81]
Photoplethysmography	18 (26.9)	[17,21,22,31,35,39,48,50,56,59,62,67,68,70,75,76,79,81]
Thermometer	12 (17.9)	[17,27,35,39,44,48,62,63,75,76,79,81]
Electrocardiogram	11 (16.4)	[16,18,19,32-34,43,44,61,73,74]
Gyroscope	9 (13.4)	[28,31,36,48,55,68,71,72,77]
Electromyogram	6 (9.0)	[30,36,43,44,57,77]
Other (n<3)^b^	8 (11.9)	[16,27,31,43,44,48,63,69]
Sensing technology		
Opportunistic	63 (94.0)	[15-33,35,37-39,41,43-67,69-81]
Participatory	1 (1.5)	[42]
Not reported	3 (4.5)	[34,36,40]

a Biosignals represent the physiological metrics measured by wearable devices. Sensor types refer to the specific technologies embedded in wearable devices.

b Include different sensors of wearable devices that add up to less than three studies.

### Features of AI

Table 5 shows a problem-solving method, type of AI algorithm, and proposed function in all included studies (n=67). This finding indicates that classification was the main method used in all studies (100%). Support vector machines (SVMs; n=28, 41.8%) are the most widely used AI algorithms, followed by random forests (n=14, 20.9%), convolutional neural networks (CNNs; n=10, 14.9%), boosting models, and logistic regression (n=8, 11.9%). Less common methods include deep neural networks (n=7, 10.4%), K-nearest neighbors (n=6, 9%), repetition neural networks (n=9, 9%), naive Bayes (n=4, 6%), and multilayer perceptron (n=4, 6%). A small number of studies (n=5, 7.5%) used other algorithms, but 3% (n=2) did not report the algorithms used. As for the purpose of using AI, most studies focused on detecting epilepsy (n=54, 80.6%), while the target prediction rate was 22.4% (n=15; see Multimedia Appendix 5).

**Table 5.** Artificial intelligence (AI) approaches and algorithms used (n=67 studies).

Features	Studies, n (%)	References
Problem-solving approaches		
Classification	67 (100)	[15-81]
AI algorithms		
Support vector machines	28 (41.8)	[15,27,29-32,35,38,40,42,47,49,52-55,57,58,61,63,67-69,71,73,75,78,80]
Random forest	14 (20.9)	[15,23,33,40,45,46,49,55,70,73-75,77,78]
Convolutional networks	10 (14.9)	[25,36,39,41,60,64,65,72,76,79]
Boosting models	8 (11.9)	[18,21,22,26,48,51,75,81]
Logistic regression	8 (11.9)	[15,35,52,67,70,74,75,78]
Deep neural networks	7 (10.4)	[19,20,24,28,52,76]
K-nearest neighbors	6 (9.0)	[20,37,45,49,67,75]
Recurrent neural networks	6 (9.0)	[16,46,56,62,70,76]
Naive Bayes	4 (6.0)	[17,55,67,75]
Multilayer perceptron	4 (6.0)	[40,59,67,71]
Other (n<3)^a^	5 (7.5)	[35,40,43,44,52]
Not reported	2 (3.0)	[34,66]
Aim of AI algorithms		
Detection	54 (80.6)	[15,16,18-26,28-38,40,42-46,48-54,57-61,63,65-69,71,73-75,77-80]
Prediction	15 (22.4)	[17,19,26,27,39,41,47,55,56,62,64,70,72,76,81]

a Include different AI algorithms that add up to less than three studies.

Table 6 summarizes the source and type of data, the nature of input signals transmitted into the AI model, and the number of features used in the included studies (n=67). Closed-source data was the most frequently used (n=44, 65.7%), and 28.4% (n=19) of studies used open-source data. The majority of the data (n=64, 95.5%) originated from wearable devices, while only 3% (n=2) was derived from non–wearable device data. In terms of data input to AI, the most common measurement is activity measures (n=34, 50.7%), followed by brain activity (n=24, 35.8%), electrodermal activity (n=21, 31.3%), and cardiovascular measurement (n=17, 25.4%). A few studies included other data inputs, such as electrocardiography (n=11, 16.4%), skin temperatures (n=10, 14.9%), electromyography (n=7, 10.4%), and blood oxygen saturation (n=2, 3.0%). While 1.5% (n=1) have not reported input types, 37.3% (n=25) of the articles have reported the number of features extracted from AI models that range from 2 to 1680, with an average of 111.7 (SD 329.4) features. Multimedia Appendix 6 presents the data characteristics and input features utilized in the AI models of each included study.

**Table 6.** Data characteristics and input features used in artificial intelligence (AI) models (n=67 studies).

Features	Studies, n (%)	References
Data source		
Closed source	44 (65.7)	[15,17,21,22,24,25,27-30,32,34,35,38,39,43-51,54,56-58,60,62,63,66-71,75-79,81]
Open source	19 (28.4)	[18-20,23,26,31,33,37,41,52,53,55,59,64,65,72-74,80]
Not reported	4 (6.0)	[16,36,40,42]
Data types		
WD^a^-based data	64 (95.5)	[15-35,37,38,40-60,62-64,67-81]
Non-WD–based data	2 (3.0)	[65,66]
Not reported	1 (1.5)	[36]
Data input to AI algorithm		
Activity measures	34 (50.7)	[15,16,20-22,27,28,30,31,35,39,42-45,47-50,53,54,56-58,60-63,66-68,77-79]
Brain activity	24 (35.8)	[18,19,23-25,29,37,38,40-42,46,52,55,61,64,65,69,71-74,80]
Electrodermal activity	21 (31.3)	[16,17,21,22,31,35,39,42,43,45,48,56,62,63,67,68,75-77,79,81]
Cardiovascular measures	17 (25.4)	[17,21,27,31,35,39,42,48,50,56,59,62,70,75,76,79,81]
Electrocardiography	11 (16.4)	[16,19,32-34,43,44,61,72-74]
Skin temperature	10 (14.9)	[17,27,31,35,39,44,56,62,75,77]
Electromyography	7 (10.4)	[30,31,43,44,57,72,77]
Blood oxygen saturation	2 (3.0)	[27,50]
Other (n=1)^b^	3 (4.5)	[16,70]
Not reported	1 (1.5)	[36]
Number of features		
Reported	25 (37.3)	[15,17,22,23,26,28,29,31,35,38,40,43,45,48,53,58,63,70,73-75,77,78,80,81]
Not reported	42 (62.7)	[16,18-21,24,25,28,30,32-34,36,37,39,41,42,44,46,47,49-52,54-57,59-62,64-69,71,72,76,79]

a WD: wearable device.

b Included types of the data input to AI that add up to one study.

Table 7 presents the reference standards, model validation methods, and performance metrics used in the included studies (n=67). The most commonly reported reference standard was the video recording with an EEG signal (n=35, 52.2%), followed by an EEG signal only (n=16, 23.9%). Only two studies (n=2, 3%) used electrocardiogram signals as a reference, and 20.9% (n=14) did not specify a reference standard. Different methods were used to evaluate and validate model performance. The leave-one-out cross-validation was the most common (n=21, 31.3%), followed by K-fold cross-validation (n=20, 29.9%) and training-test split (n=20, 29.9%). External validation has rarely been used (n=1, 1.5%), and 10.4% (n=7) of studies have not specified their validation method. Additionally, performance metrics were most commonly reported for sensitivity (n= 54, 80.6%) and accuracy (n=28, 41.8%). Additionally, metrics for specificity (n=24, 35.8%), false alarm rate (n=22, 32.8%), and precision (n=19, 28.4%) were frequently included. Multimedia Appendix 5 provides details on the AI features utilized in each of the cited studies.

**Table 7.** Model evaluation strategies and machine learning performance metrics (n=67 studies).

Featurs	Studies, n (%)	References
Reference standard		
Video recording with EEG^a^ signals^b^	35 (52.2)	[15,17,18,21,22,24,25,28,33,35,37-40,43,45,46,48,49,51,53,54,56-58,61,63,68,71,74-77,79,81]
EEG signals	16 (23.9)	[19,23,26,41,42,52,55,59,62,64,65,69,71-73,80]
Electrocardiogram signals	2 (3)	[32,34]
Not reported	14 (20.9)	[16,20,27,30,31,36,44,47,50,60,66,67,70,78]
Model evaluation and validation		
Leave-one-out cross-validation	21 (31.3)	[21,22,24,32-34,37-39,45,46,48,53,54,56-58,61,63,73,77]
K-fold cross-validation	20 (29.9)	[15,17,25,26,28,35,40,41,48,55,59,62-64,70,74,75,79-81]
Training-test split	20 (29.9)	[16,18,19,23,27,29,31,42,47,49,50,52,61,65-67,71,72,78]
External validation	1 (1.5)	[30]
Not reported	7 (10.4)	[20,36,43,44,68,69,76]
Machine learning performance measures		
Sensitivity	54 (80.6)	[15,17,19,21-26,28-36,38-46,48-58,60-64,66-68,72-75,77,79,80]
Accuracy	28 (41.8)	[23,24,27-29,31,35,36,40,41,44,47,48,50,55,60,64-67,69-72,75,76,78,81]
Specificity	24 (35.8)	[17,19,23,24,26,29-33,35,36,41,43-46,52,53,59,60,64,74,75]
False alarm rate	22 (32.8)	[15,18,20-22,27,34,38,48,54,57,61-63,68,73,74,77-80]
Precision	19 (28.4)	[16,21,22,24,25,28,39-42,45,52,53,55,63,66,67,72]
Area under curve	10 (14.9)	[35,39,41-43,48,62,63,66,67,72]
False-positive rate	7 (10.4)	[25,37,49-51,64,79]
*F*_1_-score	11 (16.4)	[27,28,39,40,42,53,55,63,66,67,72]
Negative predictive value	3 (4.5)	[23,24,52]
Detection latency	3 (4.5)	[57,63,79]
Other (n<3)^c^	8 (11.9)	[15,17,23,26,27,33,64,70,76,78]

a EEG: electroencephalogram.

b Video recording with EEG signals refers to the combined use of EEG and video monitoring for verifying seizure events.

c Include different types of machine learning performance measures that add up to less than three studies.

## Discussion

### Principal Findings

This work analyzed 67 studies focused on wearable AI devices for epilepsy detection. A growing interest in using such devices from 2021 to 2023 was found, particularly in technologically advanced countries like India, the United States, and China. This trend underscores the increasing recognition of AI’s potential in epilepsy management. The comparable distribution of commercial (n=31, 46.3%) and noncommercial (n=31, 46.3%) wearable devices suggests developments from industry and research laboratories. Empatica and smart bands were dominant choices for activity and cardiovascular signal measurements. Also, the prevalent use of accelerometers and EEG sensors indicates their importance in improving the accuracy of seizure detection on the used devices. The use of various devices and biosignals highlights the versatility of wearable AI while also reflecting challenges such as data variability and device reliability.

The results indicated that SVMs (n=28, 41.8%), random forests (n=14, 20.9%), and CNNs (n=10, 14.9%) were the most commonly used AI models in wearable epilepsy research. SVMs were favored due to their robustness in handling high-dimensional physiological data with limited sample sizes, a common characteristic of wearable datasets. Random forests were used extensively due to their interpretability and ability to handle imbalanced datasets through ensemble learning. CNNs were widely used in research, taking advantage of time-series or image-like inputs from biosignals (ie, EEG or accelerometry), leveraging their spatial feature extraction. Such model selection is a trade-off between model complexity, interpretability, and ease of deployment on resource-constrained wearable devices. There were not many studies that utilized large-scale deep learning models, possibly due to the fact that large open datasets were scarce and energy efficiency was needed for wearable applications. It is found that studies employing ensemble methods (eg, boosting models: n=8, 11.9%) reported competitive performance. Such models could be further explored to enhance model detection accuracy.

The inclusion of multimodal data with diverse biosignals (eg, skin conductance: n=23, 34.3%; neuromuscular activity: n=8, 11.9%; respiratory measures: n=3, 4.5%) improved seizure detection rates. However, the scarcity of open-source datasets triggers the need for building comprehensive and accessible repositories. Few studies employed participatory sensing. Hence, there is an opportunity to incorporate patient feedback into data collection to enhance user engagement and personalization. Additional findings from our review reveal that smartwatches, though less common (n=7, 10.4%), have shown promising results in continuous seizure monitoring. On the other hand, EEG devices, despite being noninvasive, were often reported as less comfortable for long-term use compared to wrist-worn devices. Such a finding aligns with the need for user-centric design in medical wearables. Validation methods used for evaluating the AI models implemented in the wearable devices varied, with leave-one-out cross-validation (n=21, 31.3%) and K-fold cross-validation (n=20, 29.9%) being the most common ones. A frequent use of video EEG as a reference standard (n=35, 52.2%) was evident, which emphasizes the importance of reliable benchmarking in AI development. Most studies focused on adults (n=31%, 46.3%), with children and older participants being underrepresented. Performance metrics such as sensitivity (n=54, 80.6%), accuracy (n=28, 41.8%), and specificity (n=24, 35.8%) were widely reported, yet false alarms (n=22, 32.8%) remain a concern and indicate room for algorithmic refinement. Our review highlights critical gaps such as standardized validation, open data access, diverse demographic inclusion, and participatory sensing. Addressing these will enhance the clinical reliability and patient-centricity of AI-powered epilepsy management solutions.

### Clinical Implications

The findings of this scoping review have significant clinical practice and real-world implications for the application of AI-based wearable technology in epilepsy treatment. Increased use of noninvasive wearable technology, particularly wrist-mounted devices such as smart bands and smartwatches, is an indication of more user-oriented continuous monitoring technology accommodated within patient lifestyles. This is a change that can improve compliance of patients, as these systems are less obtrusive and more comfortable than traditional EEG systems and enable long-term monitoring with minimal disruption to daily life. Real-time seizure detection, as demonstrated by the majority of studies reviewed (n=54, 80.6%), holds the potential for timely clinical intervention that might reduce the severity and damage of uncontrolled seizures. Early warning systems, although less established, would allow for patients and caregivers to institute preventive strategies in advance, such as rapid-acting medication or moving to a safe environment, and increase patient safety and autonomy. From an operations clinical perspective, wearable AI devices can decrease the need for prolonged hospital-based EEG monitoring, thereby cutting health care costs and burden. In addition, multimodal biosignal fusion (such as activity, cardiovascular, and electrodermal metrics) and interpretable AI models allow for the development of clinically explainable AI systems, which are a vital factor for doctor trust and uptake.

### Research and Future Directions

This review underscores some research implications for future studies on wearable AI for epilepsy management. One such direction is related to the need to explore multimodal data collection that integrates biosignals (eg, cardiovascular measures, skin conductance, and neuromuscular activity) alongside brain activity measurements. Such an approach not only enhances the accuracy of seizure prediction models but also helps to develop early warning systems. Incorporating larger datasets from diverse demographics is also essential to improve the generalizability of the developed AI models.

Future research could focus on the development of user-friendly wearable devices (eg, smartwatches and wristbands) due to their higher user acceptance. Furthermore, devices capable of predicting seizures, besides the detection or monitoring tasks, will be particularly valuable. This could be achieved with wearable sensors equipped with advanced AI algorithms (ie, deep learning and large AI models). Providing timely alerts can allow patients and caregivers to take preventive measures that reduce seizure-related risks. One research direction could be focused on the development of large-scale deep learning frameworks that provide inference on the cloud without compromising device performance. Researchers could also focus on enhancing model transparency and interpretability to facilitate clinical adoption. Developing lightweight deep learning AI models, optimized for mobile and wearable devices, can provide real-time processing and large-scale data analysis that is essential for enhancing epilepsy management. Such models could be coupled with edge computing to minimize latency. One important implication is the need for more collaboration between AI developers, clinicians, and device manufacturers. Interdisciplinary approaches can ensure that AI algorithms are clinically relevant and can address the issues of false alarms and patient discomfort. Collaborative efforts can also lead to standardized protocols for data collection, validation, and sharing.

The ethical and regulatory aspects of AI in epilepsy care should be addressed. Future implications should explore frameworks for data privacy and security and algorithmic fairness. Integrating patient feedback into the design and implementation of wearable AI systems can also enhance their usability and acceptance. Security and privacy of the information are priorities due to the continuous collection and transmission of sensitive physiological data, often in real time. Wearables that record biosignals such as EEG, electrocardiogram, or electrodermal activity can unwittingly reveal aspects of a user’s neurological health, activity, and location. Lacking adequate encryption, secure storage centers in the cloud, and stringent access controls, these data streams may be vulnerable to breaches or misuse. They must comply with region-specific data privacy laws, such as the General Data Protection Regulation for the European region or the Health Insurance Portability and Accountability Act for the United States, and ensure that informed consent procedures are open and comprehensive. Regulator wise, the approval of AI-enabled wearables remains a moving target. The majority of existing frameworks, such as the Food and Drug Administration’s Digital Health Software Precertification Program or the European Medical Device Regulation, remain in catch-up mode with the complexity of dynamic, always-learning AI models. These kinds of systems oftentimes fall under Software as a Medical Device, which requires stringent validation for safety, efficacy, and risk management. Features for real-time seizure prediction or detection, especially those having therapeutic or alerting implications, will likely require follow-up clinical trials and postmarket surveillance to be approved by regulators.

### Comparison to Prior Work

Compared to past reviews of seizure detection and wearable technology, this scoping review has a broader and more targeted synthesis at the intersection of AI, epilepsy, and noninvasive wearable technology. Past studies were prone to focus narrowly on EEG-based systems or novel machine learning techniques without considering the full gamut of wearable biosignals or device forms. For instance, the work by Li et al [9] provided a general overview of wearable devices but did not rigorously review the AI algorithms or validation strategies implemented in epilepsy therapy. The work by Beniczky et al [6] dealt with clinical practice guidelines without indicating the technical specifics of AI methods. Other works (eg, [10,13]) were primarily concerned with EEG-based seizure detection without regard to multimodal sensing or real-time deployment limitations. This work, on the other hand, brings together clinical and technical perspectives; includes both commercial and noncommercial devices; maps validation strategies, availability of datasets, and demographic consistency; and thus offers a more extensive and realistic foundation for the development of wearable AI for the treatment of epilepsy.

### Strengths and Limitations

This review represents a systematic and methodologically rigorous synthesis of the wearable AI technology for the epilepsy field. Adhering to PRISMA-ScR guidance and systematically searching across six multidisciplinary databases, it includes a wide range of both engineering and clinical studies. Its coverage of more than one biosignal modality, AI algorithm, and device type represents an entire picture of the field. Moreover, the review highlights key research needs and provides unambiguous guidance for future development, such that it is a highly useful publication for researchers, clinicians, and device developers alike.

While this scoping review offers valuable insights into the landscape of wearable AI technologies for epilepsy, some limitations are to be considered when interpreting the findings. First, we only looked at wearable AI solutions in epilepsy, limiting generalizability to other neurological diseases that might be managed with similar technologies. Second, the majority of included studies were focused on detecting rather than predicting seizures. The resulting research imbalance limits us from forming strong conclusions about the performance of predictive models within the real-world environment. Third, our exclusion of in-body and near-body wearable devices, and nonwearable or handheld solutions, limits the review scope and possibly overlooks alternatives in epilepsy treatment. Fourth, we only looked at English articles after 2013, which may have led to overlooking prior or non–English-language studies on the issue. Fifth, most of the included studies used small sample sizes, closed-source data, and differing validation approaches. These render their findings less reproducible, weaker, and less clinically relevant. Lastly, by restricting the review to noninvasive wearables, we excluded studies using hybrid systems that may combine wearables with environmental sensors or mobile apps, which may offer a prolonged seizure management solution.

### Conclusions

This review highlights the transformative potential of wearable AI technologies in epilepsy management, particularly in the detection and prediction of epileptic seizures. While machine learning algorithms like SVMs, random forests, and CNNs have shown notable performance, challenges remain regarding limited access to open-source datasets, inconsistent validation methods, and small sample sizes. Addressing these issues through open data sharing, standardized evaluation frameworks, and large-scale clinical validation will be critical.

The study emphasizes the importance of developing lightweight AI models optimized for real-time processing on wearable devices, while also exploring cloud-based solutions for complex analysis. Multimodal data integration—including physiological, behavioral, and contextual signals—can significantly enhance predictive performance and reliability, paving the way for proactive and personalized epilepsy care.

Future research should prioritize ethical considerations such as data privacy, informed consent, and algorithmic transparency, alongside regulatory compliance to ensure safe deployment in clinical settings. Cross-sector collaboration among AI developers, clinicians, engineers, and regulators will be essential to deliver trustworthy, user-centered wearable solutions that can meaningfully reduce the burden of epilepsy worldwide.

## Supplementary material

10.2196/73593 Multimedia Appendix 1 Search strategy.

10.2196/73593 Multimedia Appendix 2 Data extraction form.

10.2196/73593 Multimedia Appendix 3 Characteristics of each included study.

10.2196/73593 Multimedia Appendix 4 Features of wearable devices and sensors.

10.2196/73593 Multimedia Appendix 5 Features of artificial intelligence algorithms.

10.2196/73593 Multimedia Appendix 6 Features of data used in artificial intelligence algorithms.

10.2196/73593 Checklist 1 PRISMA-ScR checklist.

## Abbreviations

AI: artificial intelligence
CNN: convolutional neural network
EEG: electroencephalography
PRISMA-ScR: Preferred Reporting Items for Systematic Reviews and Meta-Analyses Extension for Scoping Reviews
SVM: support vector machine
