# Supplementary material for: Wearable Artificial Intelligence for Epilepsy: Scoping Review
Source: J Med Internet Res. 2025 Oct 31;27:e73593. doi: 10.2196/73593 (PMC12578435; doi:10.2196/73593)
Supplement: Multimedia Appendix 1 [file jmir-v27-e73593-s001.docx]

**Appendix 2: Search strategy**

Database(s): **Ovid MEDLINE(R) ALL**1946 to December 07, 2023
Search Strategy:

| **#** | **Searches** | **Results** |
| --- | --- | --- |
| 1 | exp epilepsy/ | 127299 |
| 2 | Epilep*.tw. | 163245 |
| 3 | exp seizure/ | 74772 |
| 4 | seizure*.tw. | 144715 |
| 5 | 1 or 2 or 3 or 4 | 268931 |
| 6 | exp artificial intelligence/ | 184425 |
| 7 | "Artificial Intelligence".tw. | 35920 |
| 8 | exp Machine Learning/ | 62490 |
| 9 | "Machine Learning".tw. | 88751 |
| 10 | exp Deep Learning/ | 17484 |
| 11 | "Deep Learning".tw. | 48629 |
| 12 | "Supervised Learning".tw. | 5185 |
| 13 | "Unsupervised Learning".tw. | 2247 |
| 14 | "Semi-supervised Learning".tw. | 918 |
| 15 | "Reinforcement Learning".tw. | 5835 |
| 16 | "Decision Tree*".tw. | 15402 |
| 17 | "K-Nearest Neighbor*".tw. | 5336 |
| 18 | "Support vector machine*".tw. | 26257 |
| 19 | "Recurrent Neural Network*".tw. | 4288 |
| 20 | "Convolutional Neural Network*".tw. | 24337 |
| 21 | "Artificial neural network*".tw. | 17574 |
| 22 | "Deep Neural Network*".tw. | 9494 |
| 23 | "Naïve Bayes".tw. | 9 |
| 24 | "Naive Bayes".tw. | 3300 |
| 25 | "Bayesian Networks".tw. | 1535 |
| 26 | "Fuzzy Logic".tw. | 2450 |
| 27 | "K-Means".tw. | 7289 |
| 28 | "Random Forest*".tw. | 23546 |
| 29 | "Long Short-Term Memory*".tw. | 4624 |
| 30 | "Autoencoder".tw. | 2602 |
| 31 | "Boltzmann Machine".tw. | 314 |
| 32 | "Deep Belief Network*".tw. | 423 |
| 33 | "Gradient Boost*".tw. | 5927 |
| 34 | AdaBoost.tw. | 1415 |
| 35 | "Multilayer Perceptron".tw. | 2721 |
| 36 | "Ensemble learning".tw. | 1874 |
| 37 | "Generative Adversarial Network*".tw. | 3158 |
| 38 | "Transfer Learning".tw. | 5137 |
| 39 | 6 or 7 or 8 or 9 or 10 or 11 or 12 or 13 or 14 or 15 or 16 or 17 or 18 or 19 or 20 or 21 or 22 or 23 or 24 or 25 or 26 or 27 or 28 or 29 or 30 or 31 or 32 or 33 or 34 or 35 or 36 or 37 or 38 | 325797 |
| 40 | exp wearable device/ | 19443 |
| 41 | wearable*.tw. | 26598 |
| 42 | "smart watch*".tw. | 230 |
| 43 | smartwatch*.tw. | 1085 |
| 44 | "smart band*".tw. | 95 |
| 45 | smartband*.tw. | 22 |
| 46 | acceleromet*.tw. | 22495 |
| 47 | gyroscop*.tw. | 2503 |
| 48 | "inertial sensor".tw. | 1175 |
| 49 | "inertial measurement unit*".tw. | 2945 |
| 50 | headband*.tw. | 375 |
| 51 | "head band*".tw. | 75 |
| 52 | "wrist band*".tw. | 99 |
| 53 | wristband*.tw. | 800 |
| 54 | armband.tw. | 704 |
| 55 | "bracelet*".tw. | 707 |
| 56 | wristwatch.tw. | 215 |
| 57 | "multimodal biosensors".tw. | 3 |
| 58 | "wearable technology".tw. | 1159 |
| 59 | magnetometers.tw. | 810 |
| 60 | "Embrace 2".tw. | 1 |
| 61 | EDDI.tw. | 49 |
| 62 | SPEAC.tw. | 7 |
| 63 | "Epi-Care-free".tw. | 1 |
| 64 | "Night Watch".tw. | 25 |
| 65 | Brio.tw. | 16 |
| 66 | "Pulse Companion".tw. | 0 |
| 67 | BioStamp.tw. | 12 |
| 68 | Empatica.tw. | 103 |
| 69 | "Apple Watch".tw. | 319 |
| 70 | Amazfit.tw. | 6 |
| 71 | "Oura Ring".tw. | 42 |
| 72 | Vivosmart.tw. | 44 |
| 73 | 40 or 41 or 42 or 43 or 44 or 45 or 46 or 47 or 48 or 49 or 50 or 51 or 52 or 53 or 54 or 55 or 56 or 57 or 58 or 59 or 60 or 61 or 62 or 63 or 64 or 65 or 66 or 67 or 68 or 69 or 70 or 71 or 72 | 66960 |
| 74 | 5 and 39 and 73 | 108 |
| 75 | limit 74 to (english language and humans) | 82 |

Database(s): **Embase**1974 to 2023 Week 49
Search Strategy:

| **#** | **Searches** | **Results** |
| --- | --- | --- |
| 1 | exp epilepsy/ | 276739 |
| 2 | Epilep*.tw. | 232248 |
| 3 | exp seizure/ | 221300 |
| 4 | seizure*.tw. | 220338 |
| 5 | 1 or 2 or 3 or 4 | 445576 |
| 6 | exp artificial intelligence/ | 91333 |
| 7 | "Artificial Intelligence".tw. | 42952 |
| 8 | exp Machine Learning/ | 436052 |
| 9 | "Machine Learning".tw. | 104792 |
| 10 | exp Deep Learning/ | 48484 |
| 11 | "Deep Learning".tw. | 56469 |
| 12 | "Supervised Learning".tw. | 5780 |
| 13 | "Unsupervised Learning".tw. | 2511 |
| 14 | "Semi-supervised Learning".tw. | 1026 |
| 15 | "Reinforcement Learning".tw. | 6507 |
| 16 | "Decision Tree*".tw. | 21418 |
| 17 | "K-Nearest Neighbor*".tw. | 6240 |
| 18 | "Support vector machine*".tw. | 31396 |
| 19 | "Recurrent Neural Network*".tw. | 4778 |
| 20 | "Convolutional Neural Network*".tw. | 28531 |
| 21 | "Artificial neural network*".tw. | 20356 |
| 22 | "Deep Neural Network*".tw. | 10547 |
| 23 | "Naïve Bayes".tw. | 24 |
| 24 | "Naive Bayes".tw. | 4033 |
| 25 | "Bayesian Networks".tw. | 1772 |
| 26 | "Fuzzy Logic".tw. | 2957 |
| 27 | "K-Means".tw. | 9728 |
| 28 | "Random Forest*".tw. | 29009 |
| 29 | "Long Short-Term Memory*".tw. | 4674 |
| 30 | "Autoencoder".tw. | 2867 |
| 31 | "Boltzmann Machine".tw. | 347 |
| 32 | "Deep Belief Network*".tw. | 475 |
| 33 | "Gradient Boost*".tw. | 7166 |
| 34 | AdaBoost.tw. | 1706 |
| 35 | "Multilayer Perceptron".tw. | 3018 |
| 36 | "Ensemble learning".tw. | 2115 |
| 37 | "Generative Adversarial Network*".tw. | 3569 |
| 38 | "Transfer Learning".tw. | 5509 |
| 39 | 6 or 7 or 8 or 9 or 10 or 11 or 12 or 13 or 14 or 15 or 16 or 17 or 18 or 19 or 20 or 21 or 22 or 23 or 24 or 25 or 26 or 27 or 28 or 29 or 30 or 31 or 32 or 33 or 34 or 35 or 36 or 37 or 38 | 535879 |
| 40 | exp wearable device/ | 0 |
| 41 | wearable*.tw. | 28978 |
| 42 | "smart watch*".tw. | 355 |
| 43 | smartwatch*.tw. | 1307 |
| 44 | "smart band*".tw. | 99 |
| 45 | smartband*.tw. | 22 |
| 46 | acceleromet*.tw. | 28252 |
| 47 | gyroscop*.tw. | 2484 |
| 48 | "inertial sensor".tw. | 1357 |
| 49 | "inertial measurement unit*".tw. | 3112 |
| 50 | headband*.tw. | 547 |
| 51 | "head band*".tw. | 110 |
| 52 | "wrist band*".tw. | 184 |
| 53 | wristband*.tw. | 1133 |
| 54 | armband.tw. | 1286 |
| 55 | "bracelet*".tw. | 1033 |
| 56 | wristwatch.tw. | 312 |
| 57 | "multimodal biosensors".tw. | 4 |
| 58 | "wearable technology".tw. | 1471 |
| 59 | magnetometers.tw. | 810 |
| 60 | "Embrace 2".tw. | 7 |
| 61 | EDDI.tw. | 59 |
| 62 | SPEAC.tw. | 13 |
| 63 | "Epi-Care-free".tw. | 4 |
| 64 | "Night Watch".tw. | 32 |
| 65 | Brio.tw. | 44 |
| 66 | "Pulse Companion".tw. | 0 |
| 67 | BioStamp.tw. | 27 |
| 68 | Empatica.tw. | 137 |
| 69 | "Apple Watch".tw. | 477 |
| 70 | Amazfit.tw. | 8 |
| 71 | "Oura Ring".tw. | 61 |
| 72 | Vivosmart.tw. | 52 |
| 73 | 40 or 41 or 42 or 43 or 44 or 45 or 46 or 47 or 48 or 49 or 50 or 51 or 52 or 53 or 54 or 55 or 56 or 57 or 58 or 59 or 60 or 61 or 62 or 63 or 64 or 65 or 66 or 67 or 68 or 69 or 70 or 71 or 72 | 64417 |
| 74 | 5 and 39 and 73 | 194 |
| 75 | limit 74 to (english language and humans) | 187 |
| 76 | limit 75 to "remove medline records" | 76 |

Database(s): **EBSCO (APA PsychInfo)**

| **#** | **Searches** | **Results** |
| --- | --- | --- |
| 1 | MA epilepsy OR MA seizure OR AB ( epilep* OR seizure* ) | 59,527 |
| 2 | "MA artificial intelligence OR MA machine learning OR MA deep learning OR AB ( "Artificial Intelligence" OR "Machine Learning" OR "Deep Learning" OR "Supervised Learning" OR "Unsupervised Learning" OR "Semi-supervised Learning" OR "Reinforcement Learning" OR "Decision Tree*" OR "K-Nearest Neighbor*" OR "Support vector machine*" OR "Recurrent Neural Network*" OR "Convolutional Neural Network*" OR "Artificial neural network*" OR "Deep Neural Network*" OR "Naïve Bayes" OR "Naive Bayes" OR "Bayesian  Networks" OR "Fuzzy Logic" OR "K-Means" OR "Random Forest*" OR "Long Short-Term Memory*" OR "Autoencoder" OR "Boltzmann Machine" OR "Deep Belief Network*" OR "Gradient Boost*" OR AdaBoost OR "Multilayer Perceptron" OR "Ensemble learning" OR "Generative Adversarial Network*" OR "Transfer Learning") | 34,809 |
| 3 | MA wearable device OR AB ( wearable* OR "smart watch*" OR smartwatch* OR "smart band*" OR smartband* OR acceleromet* OR gyroscop* OR "inertial sensor" OR "inertial measurement unit*" OR headband* OR "head band*" OR "wrist band*" OR wristband* OR armband OR "bracelet*" OR wristwatch OR "multimodal biosensors" OR "Wearable technology" OR accelerometers OR gyroscopes OR magnetometers OR "Embrace 2" OR EDDI OR SPEAC OR "Epi-Care-free" OR "Night Watch" OR Brio OR "Pulse Companion" OR BioStamp OR Empatica OR “Apple Watch” OR Amazfit OR “Oura Ring” OR Vivosmart ) | 7,738 |
| 4 | S1 AND S2 AND S3 | 6 |

Database(s): **CINHAL**

| **#** | **Searches** | **Results** |
| --- | --- | --- |
| 1 | MA epilepsy OR MA seizure OR AB ( epilep* OR seizure* ) | 44,412 |
| 2 | "MA artificial intelligence OR MA machine learning OR MA deep learning OR AB ( "Artificial Intelligence" OR "Machine Learning" OR "Deep Learning" OR "Supervised Learning" OR "Unsupervised Learning" OR "Semi-supervised Learning" OR "Reinforcement Learning" OR "Decision Tree*" OR "K-Nearest Neighbor*" OR "Support vector machine*" OR "Recurrent Neural Network*" OR "Convolutional Neural Network*" OR "Artificial neural network*" OR "Deep Neural Network*" OR "Naïve Bayes" OR "Naive Bayes" OR "Bayesian  Networks" OR "Fuzzy Logic" OR "K-Means" OR "Random Forest*" OR "Long Short-Term Memory*" OR "Autoencoder" OR "Boltzmann Machine" OR "Deep Belief Network*" OR "Gradient Boost*" OR AdaBoost OR "Multilayer Perceptron" OR "Ensemble learning" OR "Generative Adversarial Network*" OR "Transfer Learning") | 41,708 |
| 3 | MA wearable device OR AB ( wearable* OR "smart watch*" OR smartwatch* OR "smart band*" OR smartband* OR acceleromet* OR gyroscop* OR "inertial sensor" OR "inertial measurement unit*" OR headband* OR "head band*" OR "wrist band*" OR wristband* OR armband OR "bracelet*" OR wristwatch OR "multimodal biosensors" OR "Wearable technology" OR accelerometers OR gyroscopes OR magnetometers OR "Embrace 2" OR EDDI OR SPEAC OR "Epi-Care-free" OR "Night Watch" OR Brio OR "Pulse Companion" OR BioStamp OR Empatica OR “Apple Watch” OR Amazfit OR “Oura Ring” OR Vivosmart ) | 14,379 |
| 4 | S1 AND S2 AND S3 | 3 |

| **Database** | **Query** | **Results** |
| --- | --- | --- |
| **Scopus** | TITLE-ABS-KEY ( ( epilep* OR seizure* ) ) AND TITLE-ABS-KEY ( ( "Artificial Intelligence" OR "Machine Learning" OR "Deep Learning" OR "Supervised Learning" OR "Unsupervised Learning" OR "Semi-supervised Learning" OR "Reinforcement Learning" OR "Decision Tree*" OR "K-Nearest Neighbor*" OR "Support vector machine*" OR "Recurrent Neural Network*" OR "Convolutional Neural Network*" OR "Artificial neural network*" OR "Deep Neural Network*" OR "Na&#239;ve Bayes" OR "Naive Bayes" OR "Bayesian Networks" OR "Fuzzy Logic" OR "K-Means" OR "Random Forest*" OR "Long Short-Term Memory*" OR "Autoencoder" OR "Boltzmann Machine" OR "Deep Belief Network*" OR "Gradient Boost*" OR adaboost OR "Multilayer Perceptron" OR "Ensemble learning" OR "Generative Adversarial Network*" OR "Transfer Learning" ) ) AND TITLE-ABS-KEY ( ( wearable* OR "smart watch*" OR smartwatch* OR "smart band*" OR smartband* OR acceleromet* OR gyroscop* OR "inertial sensor" OR "inertial measurement unit*" OR headband* OR "head band*" OR "wrist band*" OR wristband* OR armband OR "bracelet*" OR wristwatch OR "multimodal biosensors" OR "Wearable technology" OR magnetometers OR "Embrace 2" OR eddi OR speac OR "Epi-Care-free" OR "Night Watch" OR brio OR "Pulse Companion" OR biostamp OR empatica OR "Apple Watch" OR amazfit OR "Oura Ring" OR vivosmart ) ) AND ( LIMIT-TO ( LANGUAGE , "English" ) ) AND ( EXCLUDE ( EXACTKEYWORD , "Nonhuman" ) ) | 341 |
| **IEEE Xplore** | ("Abstract":Epilep* OR "Abstract":seizure*) AND ("Abstract":"Artificial Intelligence" OR "Abstract":"Machine Learning" OR "Abstract":"Deep Learning" OR "Abstract":"Supervised Learning" OR "Abstract":"Unsupervised Learning" OR "Abstract":"Semi-supervised Learning" OR "Abstract":"Reinforcement Learning" OR "Abstract":"Decision Tree" OR "Abstract":"K-Nearest Neighbor" OR "Abstract":"Support vector machine" OR "Abstract":"Recurrent Neural Network" OR "Abstract":"Convolutional Neural Network" OR "Abstract":"Artificial neural network" OR "Abstract":"Deep Neural Network" OR "Abstract":"Naïve Bayes" OR "Abstract":"Naive Bayes" OR "Abstract":"Bayesian Networks" OR "Abstract":"Fuzzy Logic" OR "Abstract":"K-Means" OR "Abstract":"Random Forest" OR "Abstract":"Long Short-Term Memory" OR "Abstract":"Autoencoder" OR "Abstract":"Boltzmann Machine" OR "Abstract":"Deep Belief Network" OR "Abstract":"Gradient Boost*" OR "Abstract":AdaBoost OR "Abstract":"Multilayer Perceptron" OR "Abstract":"Ensemble learning" OR "Abstract":"Generative Adversarial Network" OR "Abstract":"Transfer Learning") AND ("Abstract":wearable* OR "Abstract":"smart watch" OR "Abstract":smartwatch OR "Abstract":"smart band" OR "Abstract":smartband* OR "Abstract":acceleromet* OR "Abstract":gyroscop OR "Abstract":"inertial sensor" OR "Abstract":"inertial measurement unit" OR "Abstract":headband* OR "Abstract":"head band" OR "Abstract":"wrist band" OR "Abstract":wristband* OR "Abstract":armband OR "Abstract":"bracelet*" OR "Abstract":wristwatch OR "Abstract":"multimodal biosensors" OR "Abstract":"Wearable technology" OR "Abstract":magnetometers OR "Abstract":"Embrace 2" OR "Abstract":EDDI OR "Abstract":SPEAC OR "Abstract":"Epi-Care-free" OR "Abstract":"Night Watch" OR "Abstract":Brio OR "Abstract":"Pulse Companion" OR "Abstract":BioStamp OR "Abstract":Empatica OR "Abstract":“Apple Watch” OR "Abstract": Amazfit OR "Abstract":“Oura Ring” OR "Abstract":Vivosmart) | 107 |
| **ACM Digital library** | [[Abstract: epilep*] OR [Abstract: seizure*]] AND [[Abstract: "artificial intelligence"] OR [Abstract: "machine learning"] OR [Abstract: "deep learning"] OR [Abstract: "supervised learning"] OR [Abstract: "unsupervised learning"] OR [Abstract: "semi-supervised learning"] OR [Abstract: "reinforcement learning"] OR [Abstract: "decision tree*"] OR [Abstract: "k-nearest neighbor*"] OR [Abstract: "support vector machine*"] OR [Abstract: "recurrent neural network*"] OR [Abstract: "convolutional neural network*"] OR [Abstract: "artificial neural network*"] OR [Abstract: "deep neural network*"] OR [Abstract: "naïve bayes"] OR [Abstract: "naive bayes"] OR [Abstract: "bayesian networks"] OR [Abstract: "fuzzy logic"] OR [Abstract: "k-means"] OR [Abstract: "random forest*"] OR [Abstract: "long short-term memory*"] OR [Abstract: "autoencoder"] OR [Abstract: "boltzmann machine"] OR [Abstract: "deep belief network*"] OR [Abstract: "gradient boost*"] OR [Abstract: adaboost] OR [Abstract: "multilayer perceptron"] OR [Abstract: "ensemble learning"] OR [Abstract: "generative adversarial network*"] OR [Abstract: "transfer learning"]] AND [[Abstract: wearable*] OR [Abstract: "smart watch*"] OR [Abstract: smartwatch*] OR [Abstract: "smart band*"] OR [Abstract: smartband*] OR [Abstract: acceleromet*] OR [Abstract: gyroscop*] OR [Abstract: "inertial sensor"] OR [Abstract: "inertial measurement unit*"] OR [Abstract: headband*] OR [Abstract: "head band*"] OR [Abstract: "wrist band*"] OR [Abstract: wristband*] OR [Abstract: armband] OR [Abstract: "bracelet*"] OR [Abstract: wristwatch] OR [Abstract: "multimodal biosensors"] OR [Abstract: "wearable technology"] OR [Abstract: magnetometers] OR [Abstract: "embrace 2"] OR [Abstract: eddi] OR [Abstract: speac] OR [Abstract: "epi-care-free"] OR [Abstract: "night watch"] OR [Abstract: brio] OR [Abstract: "pulse companion"] OR [Abstract: biostamp] OR [Abstract: empatica] OR [Abstract: "apple watch"] OR [Abstract: amazfit] OR [Abstract: "oura ring"] OR [Abstract: vivosmart]] | 14 |
| **Google Scholar** | (Epilep* OR seizure*) AND ("Artificial Intelligence" OR "Machine Learning" OR "Deep Learning" OR "Supervised Learning" OR "Unsupervised Learning" OR "Semi-supervised Learning" OR "Reinforcement Learning" OR "Decision Tree*" OR "K-Nearest Neighbor*" OR "Support vector machine*" OR "Recurrent Neural Network*" OR "Convolutional Neural Network*" OR "Artificial neural network*" OR "Deep Neural Network*" OR "Naïve Bayes" OR "Naive Bayes" OR "Bayesian Networks" OR "Fuzzy Logic" OR "K-Means" OR "Random Forest*" OR "Long Short-Term Memory*" OR "Autoencoder" OR "Boltzmann Machine" OR "Deep Belief Network*" OR "Gradient Boost*" OR AdaBoost OR "Multilayer Perceptron" OR "Ensemble learning" OR "Generative Adversarial Network*" OR "Transfer Learning" ) AND (wearable* OR "smart watch*" OR smartwatch* OR "smart band*" OR smartband* OR acceleromet* OR gyroscop* OR "inertial sensor" OR "inertial measurement unit*" OR headband* OR "head band*" OR "wrist band*" OR wristband* OR armband OR "bracelet*" OR wristwatch OR "multimodal biosensors" OR "Wearable technology" OR magnetometers OR "Embrace 2" OR EDDI OR SPEAC OR "Epi-Care-free" OR "Night Watch" OR Brio OR "Pulse Companion" OR BioStamp OR Empatica OR “Apple Watch” OR Amazfit OR “Oura Ring” OR Vivosmart ) | 100 |
