# Supplementary material for: Wearable Artificial Intelligence for Epilepsy: Scoping Review
Source: J Med Internet Res. 2025 Oct 31;27:e73593. doi: 10.2196/73593 (PMC12578435; doi:10.2196/73593)
Supplement: Multimedia Appendix 2 [file jmir-v27-e73593-s002.docx]

**Appendix 3 Data extraction form**

| **Extracted data** | **Definition** |
| --- | --- |
| **Study Characteristics** | |
| Author | The first author of the study. |
| Year of publication | The year in which the study was published. |
| Country of publication | The country where the study was published. First author or corresponding author country |
| Type of publication | The venue where the study was published: peer-reviewed journal articles, dissertations, or conference proceedings |
| **Characteristics of Participants** | |
| Number of participants | What is the number of participants (Patients) from which the data was collected? |
| Participants group | Which age group do the participants (children <18, adult 18-65, elderly >65) in research belong from? |
| Mean age (range) | What is the mean/range age of the participants? |
| Female percentage | What is the female percentage of the participants? This would be either explicitly mentioned if not then calculate it if separately mentioned each gender distribution |
| **Wearable devices characteristics** | |
| Name of the wearable device | What is the name of the wearable device (e.g., Fitbit, Empatica, Apply Watch, ActiWatch, etc..)? |
| Status of the wearable device | Is the wearable device a prototype (non-commercial) or is it an already available commercial device (e.g., Fitbit, apple watch)? |
| Type of the wearable device | In which form is the wearable device available (e.g., smart band, smart watch, smart glasses, smart clothes, smart socks, smart shoes, etc.)? |
| Placement of the wearable device | Where the wearable device is worn during the experiment in paper or normally (wrist, chest, head, ears, forehead, eyes, fingers, foot, etc..)? |
| Measured biosignals | What are the biosignals measured by the wearable device (heart rate, EEG, ECG, step counts, body temperature, blood pressure, etc..)? |
| Sensors | What are the sensors embedded in the wearable device (Photoplethysmogram, accelerator, Gyroscope)? For commercial check their official website if not mentioned. |
| Sensing Technology | Does the wearable device collect the data with the user’s input (i.e., manually adding data; participatory approach) or without the user’s input (i.e., automatic mechanisms for collecting data; opportunistic approach)? |
| **AI Characteristics** | |
| Problem solving approaches | What is the problem-solving approach that the algorithm follows (Classification, regression)? |
| AI algorithm used | What are the AI algorithms/models (e.g., RF, SVM, ANN, CNN, RNN, DNN, k-NN, MLP, DBN, DBM, DPN BN, CRT, DT, LASSO, LR, MFA, MLR, MDL, NB, NN, NSC, RBFN) used in the paper? |
| Aim of AI algorithm | What was the algorithm used for (detection { something that will happen now} or prediction {Something that will happen in future in 2-3 mins or more})? |
| Data sources | What is the source of data that was used for developing the algorithms ( Open Access {Available online easily accessible my anyone} or Closed Access { collected by authors themselves or used from some previous studies that can't be used by anyone}? |
| Type of data | What is the type of data (e.g., WD-based data, self-reported data, non-WD-based data) that was used for developing the algorithm? |
| Data input | What is the data that was used for developing the algorithm? |
| Reference standard (Gold standard test) | How the actual status (e.g., diagnosis) of the user was confirmed (questionnaire (PHQ-9), interview, test etc..)? |
| Type of validation | What is the approach that was used to validate the developed algorithm (e.g., Training-test split, K-fold cross-validation, Nested Cross-Validation, Leave One Out cross-validation, Apparent validation, external validation)? |
| Performance measures used | What are the measures used to assess the performance of the algorithm (accuracy, sensitivity (recall), specificity, precision, AUC, etc...)? |
