# Supplementary material for: Wearable Artificial Intelligence for Epilepsy: Scoping Review
Source: J Med Internet Res. 2025 Oct 31;27:e73593. doi: 10.2196/73593 (PMC12578435; doi:10.2196/73593)
Supplement: Multimedia Appendix 3 [file jmir-v27-e73593-s003.docx]

**Multimedia Appendix 4: Characteristics of each included study**

| **Author [Ref]** | **Publication type** | **Country** | **Sample size** | **Mean Age** | **Female %** | **Participants group** |
| --- | --- | --- | --- | --- | --- | --- |
| Agrahri [18] | Conference Paper | India | 79 | NR | NR | NR |
| Ahmed [19] | Conference Paper | Pakistan | 4 | NR | NR | NR |
| Al-Bakri [20] | Conference Paper | United States | 3 | NR | NR | NR |
| Al-Hussaini [21] | Journal Article | United States | 73 | NR | NR | NR |
| Baghersalimi [22] | Journal Article | Switzerland | 249 | NR | 51 | Elderly |
| Borujeny [23] | Journal Article | Iran | 3 | NR | NR | Adults and Elderly |
| Bottcher [24] | Journal Article | Germany | 9 | 45 | 44 | Children, Adults and Elderly |
| Bottcher [25] | Journal Article | Germany | 10 | 32.7 | 40 | Children, Adults and Elderly |
| Buettner [26] | Conference Paper | Germany | 400 | NR | NR | NR |
| Burelo [27] | Journal Article | Switzerland | 12 | 8.2 | 33 | Children and adolescents |
| Clarke [28] | Journal Article | Australia | 103 | 28 | 66.7 | Children and Adults |
| Coşgun [29] | Journal Article | Turkey | 10 | NR | NR | Adults |
| Dhoot [30] | Conference Paper | India | NR | NR | NR | Children |
| Dong [31] | Journal Article | China | 12 | NR | 28 | Children and Adults |
| dos Sutantos [32] | Journal Article | Indonesia | 3 | 39 | 67 | Adult |
| Escobar Cruz [33] | Conference Paper | Colombia | 35 | NR | NR | NR |
| Fawzy [34] | Conference Paper | Egypt | 19 | NR | NR | NR |
| Forooghifar [35] | Conference Paper | Switzerland | 7 | NR | NR | Adults |
| Forooghifar [36] | Journal Article | Switzerland | 30 | NR | NR | Adults |
| Forooghifar [37] | Conference Paper | Switzerland | 43 | NR | NR | Adults |
| G [38] | Journal Article | India | 11 | NR | 45.5 | NR |
| Ge [39] | Journal Article | China | 13 | 5.7 | 69 | Children |
| Glaba [40] | Journal Article | Poland | 19 | 22 | 63 | Adults |
| Gu [41] | Journal Article | Belgium | 12 | 36 | 50 | Adults |
| Guo [42] | Conference Paper | United States | 64 | NR | NR | NR |
| Guo [43] | Conference Paper | China | NR | NR | NR | NR |
| Gupta [44] | Conference paper | India | NR | NR | NR | NR |
| Hakkem [45] | Conference paper | India | NR | NR | NR | NR |
| Hamlin [46] | Journal article | United States | 15 | 36.17 | 33.30 | Adults |
| Hassan [47] | Journal article | Kenya | 111 | NR | 49 | Children, Adults and Elderly |
| Heldberg [48] | Conference paper | Germany | 8 | NR | NR | NR |
| Huang [49] | Conference paper | Sweden | NR | NR | NR | NR |
| Jeyabharathi [50] | Journal Article | India | NR | NR | NR | NR |
| Jiang [51] | Journal Article | China | 99 | 38.65 | NR | Children |
| Johansson [52] | Journal Article | Sweden | 75 | NR | 63 | Adults and Elderly |
| Khan [53] | Conference Paper | UAE | NR | NR | NR | NR |
| Kok [54] | Journal Article | United Kingdom | 15 | NR | 27 | Adults |
| Kueh [55] | Journal Article | United Kingdom | NR | NR | NR | NR |
| Kusmakar [56] | Conference Paper | Australia | 8 | 28.37 | 38 | Adults |
| Kusmakar [57] | Journal Article | Australia | 79 | NR | NR | Adult |
| Mehta [58] | Conference Paper | India | 22 | 10 | 77 | Children and Adults |
| Meisel [59] | Journal Article | Germany | 69 | 9.62 | 41 | Children and Adults |
| Milošević [60] | Conference Paper | Belgium | 14 | NR | NR | Children |
| Milošević [61] | Journal Article | Belgium | 56 | NR | NR | Children |
| Mittlesteadt [62] | Journal Article | United States | 40 | 13.33 | 50 | Children and Adults |
| Motahar [63] | Conference Paper | Bangladesh | 1 | NR | NR | NR |
| Munch Nielsen [64] | Journal Article | Denmark | 30 | NR | 56.67 | Adults and Elderly |
| Nasseri [65] | Journal Article | United States | 6 | 37 | 50 | Adults |
| Onorati [66] | Journal Article | Italy | 69 | NR | 54 | Children and Adults |
| Prathaban [67] | Journal Article | India | 300 | NR | NR | Children and Adults |
| Qian [68] | Journal Article | China | 10 | NR | NR | Children and Adults |
| R [69] | Conference Paper | India | 50 | NR | NR | NR |
| Raj [70] | Conference Paper | India | 1 | NR | NR | NR |
| Regalia [71] | Journal Article | Italy | 135 | NR | NR | Children and Adults |
| Seethalakshmi [72] | Conference Paper | India | NR | NR | NR | NR |
| Stirling [73] | Journal Article | Australia | 11 | NR | NR | Adults |
| Sutanto [74] | Journal Article | Brazil | 33 | 32 | 63.6 | Children and Adults |
| Tian [75] | Journal Article | China | 57 | NR | NR | Children and Adults |
| Vandecasteele [76] | Journal Article | Belgium | 135 | NR | NR | NR |
| Varun [77] | Conference Paper | India | 82 | NR | NR | NR |
| Vieluf [78] | Journal Article | United States | 139 | NR | 47 | Children |
| Vieluf [79] | Journal Article | United States | 42 | 12 | 48 | Children |
| Wang [80] | Conference Paper | China | 9 | 23 | 22 | Children and Adults |
| Xianji [81] | Conference Paper | China | NR | NR | NR | NR |
| Yu [82] | Journal Article | Australia | 166 | NR | 48 | Children |
| Zhang [83] | Journal Article | Belgium | 81 | NR | NR | NR |
| Zsom [84] | Conference Paper | United States | 30 | NR | NR | NR |
