# Supplementary material for: Wearable Artificial Intelligence for Epilepsy: Scoping Review
Source: J Med Internet Res. 2025 Oct 31;27:e73593. doi: 10.2196/73593 (PMC12578435; doi:10.2196/73593)
Supplement: Multimedia Appendix 4 [file jmir-v27-e73593-s004.docx]

**Multimedia Appendix 5: Features of wearable devices (WD) and sensor**

| **Author [Ref]** | **Status of WD** | **Name of WD** | **Type of WD** | **Placement of WD** | **Measured biosignals** | **Sensors** | **Sensing technology** |
| --- | --- | --- | --- | --- | --- | --- | --- |
| Agrahri [18] | Commercial | Apple iPod Touch | Smart Band | Wrist | Activity measures | Accelerometer | Opportunistic |
| Ahmed [19] | Non-commercial | NR | Smart Band | Wrist, chest | Cardiovascular measures, skin conductance, activity measures, respiratory measures | Accelerometer, electrocardiogram, electrodermal activity, force sensitive resistor | Opportunistic |
| Al-Bakri [20] | Commercial | Empatica | Smart Band | Wrist | Skin conductance, cardiovascular measures, skin temperature | Electrodermal activity, photoplethysmography, thermometer | Opportunistic |
| Al-Hussaini [21] | Commercial | Byteflies Kit | Electrodes and patch | Ear, back | Brain activity, cardiovascular measures | Electroencephalogram, electrocardiogram | Opportunistic |
| Baghersalimi [22] | Non-commercial | e-Glass | Smart glasses | Head | Brain activity, cardiovascular measures | Electroencephalogram, electrocardiogram | Opportunistic |
| Borujeny [23] | Commercial | MICAz Motes | Wearable sensors | Arm, thigh | Activity measures | Accelerometer | Opportunistic |
| Bottcher [24] | Commercial | Empatica | Smartwatch | Wrist | Activity measures, skin conductance, cardiovascular measures | Accelerometer, electrodermal activity, photoplethysmography | Opportunistic |
| Bottcher [25] | Commercial | Empatica | Smartwatch | Wrist | Activity measures, skin conductance, cardiovascular measures | Accelerometer, electrodermal activity, photoplethysmography | Opportunistic |
| Buettner [26] | Non-commercial | NR | EEG device | Head | Brain activity | Electroencephalogram | Opportunistic |
| Burelo [27] | Non-commercial | NR | EEG device | Head | Brain activity | Electroencephalogram | Opportunistic |
| Clarke [28] | Non-commercial | NR | EEG device | Head | Brain activity | Electroencephalogram | Opportunistic |
| Coşgun [29] | NR | NR | EEG device | Head | Brain activity | Electroencephalogram | Opportunistic |
| Dhoot [30] | Non-commercial | Seizure Tracker | Wearable sensors | Ear | Skin conductance, cardiovascular measures, skin temperature, respiratory measures, activity measures, acoustic | Biometric sensor hub, thermometer, inertial measurement unit, galvanic skin response | Opportunistic |
| Dong [31] | Non-commercial | NR | Smart Band | Wrist | Activity measures, orientation | Accelerometer, gyroscope | Opportunistic |
| dos Sutantos [32] | Non-commercial | NR | EEG device | Head | Brain activity | Electroencephalogram | Opportunistic |
| Escobar Cruz [33] | Non-commercial | NR | Smart glove | Arm | Activity measures, neuromuscular activity | Accelerometer, electromyogram | Opportunistic |
| Fawzy [34] | Commercial | Empatica, Byteflies Kit and Epilog | Smart Band, wearable sensors | Wrist, Head | Activity measures, skin conductance, skin temperature, neuromuscular activity, cardiovascular measures, brain activity | Gyroscope, accelerometer, electrodermal activity, photoplethysmography, biopotential | Opportunistic |
| Forooghifar [35] | Non-commercial | SmartCardia INYU | EEG device | NR | Cardiovascular measures | Electrocardiogram | Opportunistic |
| Forooghifar [36] | Non-commercial | SmartCardia INYU | EEG device | Thoracic | Cardiovascular measures | Electrocardiogram | Opportunistic |
| Forooghifar [37] | Non-commercial | ePatch | EEG device | Thoracic | Cardiovascular measures | Electrocardiogram | NR |
| G [38] | Commercial | Empatica | Smartwatch | Wrist | Cardiovascular measures, skin conductance, activity measures, skin temperature | Photoplethysmography, electrodermal activity, accelerometer, thermometer | Opportunistic |
| Ge [39] | NR | Real-Brainhealth | NR | NR | Activity measures, orientation, skin conductance, neuromuscular activity | Accelerometer, gyroscope, electrodermal activity, electromyogram | NR |
| Glaba [40] | Non-commercial | Elmiko Digitrack | Wearable sensors | Head | Brain activity | Electroencephalogram | Opportunistic |
| Gu [41] | Non-commercial | NR | EEG device | Ear | Brain activity | Electroencephalogram | Opportunistic |
| Guo [42] | Commercial | Empatica | Smart watch | Wrist | Activity measures, cardiovascular measures, skin conductance, skin temperature | Accelerometer, electrodermal activity, photoplethysmography, thermometer | Opportunistic |
| Guo [43] | Non-commercial | NR | EEG device | NR | Brain activity | Electroencephalogram | NR |
| Gupta [44] | Commercial | NR | EEG device | Head | Brain activity | Electroencephalogram | Opportunistic |
| Hakkem [45] | Non-commercial | NR | EEG device | Wrist, Ankle | Brain activity, skin conductance, activity measures, cardiovascular measures | Electroencephalogram | Participatory |
| Hamlin [46] | Non-commercial | NR | Wearable sensors | Chest, Wrist, Arm | Cardiovascular measures, skin conductance, neuromuscular activity, activity measures, acoustic | Electrocardiogram, electromyogram, electrodermal activity, accelerometer, acoustic | Opportunistic |
| Hassan [47] | Non-commercial | NR | Wearable sensors | Wrist, Chest, Arm | Cardiovascular measures, neuromuscular activity, activity measures, skin temperature | Electrocardiogram, electromyogram, accelerometer, thermometer | Opportunistic |
| Heldberg [48] | Commercial | Empatica | Smart Band | Wrist | Skin conductance, activity measures | Electrodermal activity, accelerometer | Opportunistic |
| Huang [49] | Commercial | Byteflies Kit | Wearable sensors | NR | Brain activity | Electroencephalogram | Opportunistic |
| Jeyabharathi [50] | Non-commercial | NR | Smart watch | Wrist | Activity measures | Accelerometer | Opportunistic |
| Jiang [51] | Commercial | Microsoft Band wristband | Smart Band | Ankle | Cardiovascular measures, activity measures | Photoplethysmography, galvanic skin response, accelerometer, gyroscope, thermometer | Opportunistic |
| Johansson [52] | Commercial | Shimmer3 | Smart Band | Wrist | Activity measures | Accelerometer | Opportunistic |
| Khan [53] | Commercial | EpiPatch | Patch | Skin | Cardiovascular measures, respiratory measures, activity measures | Accelerometer, photoplethysmography | Opportunistic |
| Kok [54] | Non-commercial | NR | Wearable sensors | Neck | Acoustic | Accelerometer | Opportunistic |
| Kueh [55] | Non-commercial | NR | NR | NR | Brain activity | Electroencephalogram | Opportunistic |
| Kusmakar [56] | Non-commercial | NR | Smart Band | Wrist | Activity measures | Accelerometer | Opportunistic |
| Kusmakar [57] | Non-commercial | NR | Smart Band | Wrist | Activity measures | Accelerometer | Opportunistic |
| Mehta [58] | Commercial | NR | Wearable sensors | Head | Brain activity, cardiovascular measures, activity measures | Electroencephalogram, accelerometer, gyroscope | Opportunistic |
| Meisel [59] | Commercial | Empatica | Smart Band, Wearable sensors | Wrist, Ankle | Skin conductance, cardiovascular measures, skin temperature, activity measures | Electrodermal activity, accelerometer, photoplethysmography | Opportunistic |
| Milošević [60] | Non-Commercial | NR | Wearable sensors | Wrist, Ankle | Activity measures | Accelerometer | Opportunistic |
| Milošević [61] | Non-Commercial | NR | Smart Band, wearable sensors | Wrist, Ankle | Activity measures, neuromuscular activity | Accelerometer, electromyogram | Opportunistic |
| Mittlesteadt [62] | Commercial | Fitbit | Smart watch | Wrist | Cardiovascular measures | Accelerometer, photoplethysmography | Opportunistic |
| Motahar [63] | Non-commercial | NR | Smart Band | Wrist | Activity measures | Accelerometer | Opportunistic |
| Munch Nielsen [64] | Commercial | Enobio 8, TrackIT T4a, Faros 180, SENS Motion | Wearable sensors | Ear, Shoulder, Upper body | Brain activity, cardiovascular measures, activity measures | Accelerometer, electroencephalogram, electrocardiogram | Opportunistic |
| Nasseri [65] | Commercial | Empatica | Smart Band | Wrist | Skin conductance, activity measures, skin temperature, cardiovascular measures | Accelerometer, photoplethysmography, electrodermal activity, thermometer | Opportunistic |
| Onorati [66] | Commercial | Empatica, iCalm | Smart Band | Wrist, Arm | Activity measures, skin conductance | Electrodermal activity, ACM sensor | Opportunistic |
| Prathaban [67] | Non-commercial | ForeSeiz | Smart Band | Head | Brain activity | Electroencephalogram | Opportunistic |
| Qian [68] | Non-commercial | MCU | Wearable sensors | Head | Brain activity | Electroencephalogram | Opportunistic |
| R [69] | Commercial | Microcontroller | NR | NR | NR | Accelerometer | Opportunistic |
| Raj [70] | Non-commercial | NR | Wearable sensors | NR | Activity measures, skin conductance, cardiovascular measures | Accelerometer, electrodermal activity, photoplethysmography | Opportunistic |
| Regalia [71] | Commercial | Emptica, Embrace | Smart Band | Wrist | Skin conductance, activity measures, cardiovascular measures, skin temperature | Accelerometer, electrodermal activity, photoplethysmography, thermometer, gyroscope | Opportunistic |
| Seethalakshmi [72] | NR | NR | Wearable sensors | Wrist, Ankle | Brain activity, skin conductance, activity measures, cardiovascular measures | Electroencephalogram, electrodermal activity, ACM sensor | Opportunistic |
| Stirling [73] | Commercial | Fitbit | Smart Watch | Wrist | Cardiovascular measures, sleep measures, activity measures | Photoplethysmography, accelerometer | Opportunistic |
| Sutanto [74] | Commercial | Muse EEG headband | Smart Band (Headband) | Ear and head | Brain activity | Electroencephalogram, accelerometer, gyroscope | Opportunistic |
| Tian [75] | NR | NR | Electrodes | Head, chest, finger, hand, and wrist | Brain activity, cardiovascular measures, neuromuscular activity | Electroencephalogram, accelerometer, gyroscope | Opportunistic |
| Vandecasteele [76] | Non-commercial | Byteflies Kit | Electrodes | Ear | Brain activity, cardiovascular measures | Electroencephalogram, electrocardiogram, accelerometer | Opportunistic |
| Varun [77] | NR | NR | Wearable sensors | Ear, Head | Cardiovascular measures, skin conductance, skin temperature | Electroencephalogram, electrocardiogram | Opportunistic |
| Vieluf [78] | Commercial | Empatica | Smart Band | Wrist, Ankle | Skin conductance, cardiovascular measures | Electrodermal activity, thermometer, photoplethysmography | Opportunistic |
| Vieluf [79] | Commercial | Empatica | Smart Band | Wrist, Ankle | Activity measures, orientation, skin conductance, neuromuscular activity, skin temperature | Photoplethysmography, accelerometer, electrodermal activity, thermometer | Opportunistic |
| Wang [80] | Non-commercial | NR | Smart Band | Wrist | Activity measures | Accelerometer, gyroscope, electrodermal activity, electromyogram | Opportunistic |
| Xianji [81] | Non-commercial | NR | Smart Band | Wrist | Cardiovascular measures, activity measures, skin conductance, skin temperature | Accelerometer | Opportunistic |
| Yu [82] | commercial | Empatica | Smart Band | Wrist | Brain activity | Photoplethysmography, accelerometer, electrodermal activity, thermometer | Opportunistic |
| Zhang [83] | Commercial | Byteflies Kit | Wearable sensors | Ear, Back | Skin conductance, skin temperature, cardiovascular measures, activity measures | Electroencephalogram | Opportunistic |
| Zsom [84] | Commercial | Empatica | Smart Band | Wrist | Activity measures | Photoplethysmography, accelerometer, electrodermal activity, thermometer | Opportunistic |
