# Supplementary material for: Wearable Artificial Intelligence for Epilepsy: Scoping Review
Source: J Med Internet Res. 2025 Oct 31;27:e73593. doi: 10.2196/73593 (PMC12578435; doi:10.2196/73593)
Supplement: Multimedia Appendix 5 [file jmir-v27-e73593-s005.docx]

**Multimedia Appendix 6: Features of AI algorithms**

| **Author [Ref]** | **Problem solving approaches** | **AI algorithms** | **Aim of AI algorithms** | **Reference standard** | **Type of validation** | **ML Performance measures** |
| --- | --- | --- | --- | --- | --- | --- |
| Agrahri [18] | Classification | Support Vector Machines, Logistic Regression, Random Forest | Detection | video recording with EEG signals | K-fold Cross-Validation | Confusion Matrix, False alarm rate |
| Ahmed [19] | Classification | Recurrent Neural Networks | Detection | NR | Training-test split | Sensitivity, Precision |
| Al-Bakri [20] | Classification | Naive Bayes | Prediction | video recording with EEG signals | K-fold Cross-Validation | Sensitivity, Specificity, and Kappa Score |
| Al-Hussaini [21] | Classification | Boosting Models | Detection | video recording with EEG signals | Training-test split | Sensitivity and False alarm rate |
| Baghersalimi [22] | Classification | Deep Neural Networks | Detection, Prediction | EEG signals | Training-test split | Sensitivity, Specificity |
| Borujeny [23] | Classification | Deep Neural Networks, K-Nearest Neighbors | Detection | NR | NR | False alarm rate |
| Bottcher [24] | Classification | Boosting Models | Detection | video recording with EEG signals | Leave-One-Out Cross-Validation | Sensitivity, False alarm rate, Precision |
| Bottcher [25] | Classification | Boosting Models | Detection | video recording with EEG signals | Leave-One-Out Cross-Validation | Sensitivity, False alarm rate, Precision |
| Buettner [26] | Classification | Random Forest | Detection | EEG signals | Training-test split | Accuracy, Sensitivity, Specificity, Precision, Negative Predictive Value, Kappa score |
| Burelo [27] | Classification | Deep Neural Networks | Detection | video recording with EEG signals | Leave-One-Out Cross-Validation | Accuracy, Sensitivity, Specificity, Precision, Negative Predictive Value |
| Clarke [28] | Classification | Convolutional Networks | Detection | video recording with EEG signals | K-fold Cross-Validation | Sensitivity, False Positive Rate, Precision |
| Coşgun [29] | Classification | Boosting Models | Detection, Prediction | EEG signals | K-fold Cross-Validation | Sensitivity, Specificity, Misclassification Rate |
|  |  |  |  |  |  |  |
| Dhoot [30] | Classification | Support Vector Machines | Prediction | NR | Training-test split | Accuracy, F1 score, Misclassification Rate |
| Dong [31] | Classification | Deep Neural Networks | Detection | video recording with EEG signals | K-fold Cross-Validation | Accuracy, Sensitivity, Precision, F1 score, False Alarm Rate |
| dos Sutantos [32] | Classification | Support Vector Machines | Detection | video recording with EEG signals | Training-test split | Accuracy, Sensitivity, Specificity |
| Escobar Cruz [33] | Classification | Support Vector Machines | Detection | NR | External validation | Sensitivity, Specificity |
| Fawzy [34] | Classification | Support Vector Machines | Detection | NR | Training-test split | Accuracy, Sensitivity, Specificity |
| Forooghifar [35] | Classification | Support Vector Machines | Detection | ECG signals | Leave-One-Out Cross-Validation | Specificity, Sensitivity |
| Forooghifar [36] | Classification | Random Forest | Detection | video recording with EEG signals | Leave-One-Out Cross-Validation | Sensitivity, Specificity, Geometric mean |
| Forooghifar [37] | Classification | NR | Detection | ECG signals | Leave-One-Out Cross-Validation | Sensitivity, False Alarm Rate |
| G [38] | Classification | Support Vector Machines, Decision Trees, Logistic Regression | Detection | video recording with EEG signals | K-fold Cross-Validation | Accuracy, Sensitivity, Specificity, Area Under the Curve |
| Ge [39] | Classification | Convolutional Networks | Detection | NR | NR | Accuracy, Sensitivity and Specificity |
| Glaba [40] | Classification | K-Nearest Neighbors | Detection | video recording with EEG signals | Leave-One-Out Cross-Validation | False Positive Rate |
| Gu [41] | Classification | Support Vector Machines | Detection | video recording with EEG signals | Leave-One-Out Cross-Validation | Sensitivity, False Alarm Rate |
| Guo [42] | Classification | Convolutional Networks | Prediction | video recording with EEG signals | Leave-One-Out Cross-Validation | Area Under the Curve, Precision, Sensitivity, F1 Score |
| Guo [43] | Classification | Random Forest, Support Vector Machines, Logistic Model Tree, Multilayer Perceptron | Detection | video recording with EEG signals | K-fold Cross-Validation | Accuracy, Precision, Sensitivity, F1 Score |
| Gupta [44] | Classification | Convolutional Networks | Prediction | EEG signals | K-fold Cross-Validation | Accuracy, Sensitivity, Specificity, Precision, Area Under the Curve, |
| Hakkem [45] | Classification | Support Vector Machines | Detection | EEG signals | Training-test split | Area Under the Curve, Precision, Sensitivity, F1 Score |
| Hamlin [46] | Classification | Linear Discriminant Analysis | Detection | video recording with EEG signals | NR | Sensitivity, Specificity, Area Under the Curve |
| Hassan [47] | Classification | Fuzzy Logic Systems | Detection | NR | NR | Accuracy, Sensitivity and Specificity |
| Heldberg [48] | Classification | K-Nearest Neighbors, Random Forest | Detection | video recording with EEG signals | Leave-One-Out Cross-Validation | Sensitivity, Specificity, Precision |
| Huang [49] | Classification | Random Forest, Recurrent Neural Networks | Detection | video recording with EEG signals | Leave-One-Out Cross-Validation | Sensitivity, Specificity |
| Jeyabharathi [50] | Classification | Support Vector Machines | Prediction | NR | Training-test split | Accuracy |
| Jiang [51] | Classification | Boosting Models | Detection | video recording with EEG signals | K-fold Cross-Validation, Leave-One-Out Cross-Validation | Sensitivity, Accuracy, False Alarm Rate, Area Under the Curve |
| Johansson [52] | Classification | K-Nearest Neighbors, Support Vector Machines, Random Forest | Detection | video recording with EEG signals | Training-test split | Sensitivity, False Positive Rate |
| Khan [53] | Classification | Deep Neural Networks | Detection | NR | Training-test split | False Positive Rate, Sensitivity, Accuracy |
| Kok [54] | Classification | Boosting Models | Detection | video recording with EEG signals | Training-test split | Sensitivity, False Positive Rate |
| Kueh [55] | Classification | Logistic Model Tree, Logistic Regression, Support Vector Machines, Deep Neural Networks | Detection | EEG signals | Training-test split | Sensitivity, Specificity, Precision, Negative predictive value |
| Kusmakar [56] | Classification | Support Vector Machines | Detection | video recording with EEG signals | Leave-One-Out Cross-Validation | Sensitivity, False Alarm Rate |
| Kusmakar [57] | Classification | Support Vector Machines | Detection | video recording with EEG signals | Leave-One-Out Cross-Validation | Sensitivity, Specificity, F1 score, Precision |
| Mehta [58] | Classification | Support Vector Machines, Naive Bayes, Random Forest | Prediction | EEG signals | K-fold Cross-Validation | Accuracy, Precision, Sensitivity, F1 score |
| Meisel [59] | Classification | Recurrent Neural Networks | Prediction | video recording with EEG signals | Leave-One-Out Cross-Validation | Sensitivity |
| Milošević [60] | Classification | Support Vector Machines | Detection | video recording with EEG signals | Leave-One-Out Cross-Validation | Sensitivity, False Alarm Rate |
| Milošević [61] | Classification | Support Vector Machines | Detection | video recording with EEG signals | Leave-One-Out Cross-Validation | Sensitivity, False Alarm Rate, Detection Latency |
| Mittlesteadt [62] | Classification | Multilayer Perceptron | Detection | EEG signals | K-fold Cross-Validation | Specificity |
| Motahar [63] | Classification | Convolutional Networks | Detection | NR | Training-test split | Accuracy, Sensitivity, Specificity |
| Munch Nielsen [64] | Classification | Support Vector Machines | Detection | video recording with EEG signals | Leave-One-Out Cross-Validation | Sensitivity, False alarm rate |
| Nasseri [65] | Classification | Recurrent Neural Networks | Prediction | EEG signals | K-fold Cross-Validation | Area under the curve, Sensitivity , False alarm rate |
| Onorati [66] | Classification | Support Vector Machines | Detection | video recording with EEG signals | K-fold Cross-Validation, Leave-One-Out Cross-Validation | Sensitivity, False alarm rate, Area Under the Curve, Detection Latency, Precision, F1 score |
| Prathaban [67] | Classification | Convolutional Networks | Prediction | EEG signals | K-fold Cross-Validation | Accuracy, Sensitivity, Specificity, False Negative Rate, False Positive Rate |
| Qian [68] | Classification | Convolutional Networks | Detection | EEG signals | Training-test split | Accuracy |
| R [69] | Classification | NR | Detection | NR | Training-test split | Accuracy, Precision, Sensitivity, F1 Score |
| Raj [70] | Classification | Logistic Regression, Support Vector Machines, K-Nearest Neighbors, Naive Bayes, Multilayer Perceptron | Detection | NR | Training-test split | Accuracy, Precision, Sensitivity, F1 Score |
| Regalia [71] | Classification | Support Vector Machines | Detection | video recording with EEG signals | NR | Sensitivity and False alarm rate |
| Seethalakshmi [72] | Classification | Support Vector Machines | Detection | EEG signals | NR | Accuracy |
| Stirling [73] | Classification | Recurrent Neural Networks, Random Forest, Logistic Regression | Prediction | NR | K-fold Cross-Validation | Accuracy, Area Under the Curve, Calibration Curves, Brier Loss |
| Sutanto [74] | Classification | Support Vector Machines, Multilayer Perceptron | Detection | EEG signals | Training-test split | Accuracy |
| Tian [75] | Classification | Convolutional Networks, Deep Neural Networks | Prediction | EEG signals | Training-test split | Accuracy, Precision, Sensitivity, F1 score |
| Vandecasteele [76] | Classification | Random Forest, Support Vector Machines | Detection | EEG signals | Leave-One-Out Cross-Validation | Sensitivity, False alarm rate |
| Varun [77] | Classification | Random Forest, Logistic Regression | Detection | video recording with EEG signals | K-fold Cross-Validation | Sensitivity, Specificity, False alarm rate |
| Vieluf [78] | Classification | Logistic Regression, K-Nearest Neighbors, Random Forest, Boosting Models, Naive Bayes, Support Vector Machines | Detection | video recording with EEG signals | K-fold Cross-Validation | Accuracy, Area Under the Curve, Sensitivity, Specificity |
| Vieluf [79] | Classification | Convolutional Networks, Recurrent Neural Networks | Prediction | video recording with EEG signals | NR | Accuracy, Relative improvement over chance |
| Wang [80] | Classification | Random Forest | Detection | video recording with EEG signals | Leave-One-Out Cross-Validation | Sensitivity, False alarm rate |
| Xianji [81] | Classification | Logistic Regression, Support Vector Machines, Random Forest | Detection | NR | Training-test split | Accuracy, False negative rate, False alarm rate |
| Yu [82] | Classification | Convolutional Networks | Detection | video recording with EEG signals | K-fold Cross-Validation | Sensitivity, False Positive Rate, False alarm rate, Detection Latency |
| Zhang [83] | Classification | Support Vector Machines | Detection | EEG signals | K-fold Cross-Validation | sensitivity and False alarm rate |
| Zsom [84] | Classification | Boosting Models | Prediction | video recording with EEG signals | K-fold Cross-Validation | Accuracy |
