# Supplementary material for: Wearable Artificial Intelligence for Epilepsy: Scoping Review
Source: J Med Internet Res. 2025 Oct 31;27:e73593. doi: 10.2196/73593 (PMC12578435; doi:10.2196/73593)
Supplement: Multimedia Appendix 6 [file jmir-v27-e73593-s006.docx]

**Multimedia Appendix 7 Features of data used in AI algorithms**

| **Author [Ref]** | **Data source** | **Data types** | **Data input to AI algorithm** | **Number of features** |
| --- | --- | --- | --- | --- |
| Agrahri [18] | Closed source | WD-based data | Activity measures | 100 |
| Ahmed [19] | NR | WD-based data | Electrocardiography, Electrodermal Activity, Activity measures, and Breathing Rate | NR |
| Al-Bakri [20] | Closed source | WD-based data | Electrodermal Activity, Cardiovascular measures, Skin temperature | 16 |
| Al-Hussaini [21] | Open source | WD-based data | Brain Activity | NR |
| Baghersalimi [22] | Open source | WD-based data | Brain Activity, Electrocardiography | NR |
| Borujeny [23] | Open source | WD-based data | Activity measures | NR |
| Bottcher [24] | Closed source | WD-based data | Activity measures, Electrodermal Activity, Cardiovascular measures | NR |
| Bottcher [25] | Closed source | WD-based data | Activity measures, Electrodermal Activity | 151 |
| Buettner [26] | Open source | WD-based data | Brain Activity | 4 |
| Burelo [27] | Closed source | WD-based data | Brain Activity | NR |
| Clarke [28] | Closed source | WD-based data | Brain Activity | NR |
| Coşgun [29] | Open source | WD-based data | Brain Activity | 85 |
| Dhoot [30] | Closed source | WD-based data | Cardiovascular measures, Blood Oxygen Saturation, Skin temperature, Activity measures | NR |
| Dong [31] | Closed source | WD-based data | Activity measures | 59 |
| dos Sutantos [32] | Closed source | WD-based data | Brain Activity | NR |
| Escobar Cruz [33] | Closed source | WD-based data | Activity measures, Electromyography | NR |
| Fawzy [34] | Open source | WD-based data | Electromyography, Cardiovascular measures, Activity measures, Skin temperature, Electrodermal Activity | 23 |
| Forooghifar [35] | Closed source | WD-based data | Electrocardiography | NR |
| Forooghifar [36] | Open source | WD-based data | Electrocardiography | NR |
| Forooghifar [37] | Closed source | WD-based data | Electrocardiography | NR |
| G [38] | Closed source | WD-based data | Electrodermal Activity, Activity measures, Skin temperature, Cardiovascular measures | 58 |
| Ge [39] | NR | NR | NR | NR |
| Glaba [40] | Open source | WD-based data | Brain Activity | NR |
| Gu [41] | Closed source | WD-based data | Brain Activity | 16 |
| Guo [42] | Closed source | WD-based data | Activity measures, Cardiovascular measures, Electrodermal Activity, Skin temperature | NR |
| Guo [43] | NR | WD-based data | Brain Activity | 19 |
| Gupta [44] | Open source | WD-based data | Brain Activity | NR |
| Hakkem [45] | NR | WD-based data | Brain Activity, Electrodermal Activity, Activity measures, and Cardiovascular measures | NR |
| Hamlin [46] | Closed source | WD-based data | Electrocardiography, Electromyography, Electrodermal Activity, Activity measures | 34 |
| Hassan [47] | Closed source | WD-based data | Electrocardiography, Electromyography, Activity measures, Skin temperature | NR |
| Heldberg [48] | Closed source | WD-based data | Electrodermal Activity, Activity measures | 56 |
| Huang [49] | Closed source | WD-based data | Brain Activity | NR |
| Jeyabharathi [50] | Closed source | WD-based data | Activity measures | NR |
| Jiang [51] | Closed source | WD-based data | Cardiovascular measures, Electrodermal Activity, Activity measures, | 120 |
| Johansson [52] | Closed source | WD-based data | Activity measures | NR |
| Khan [53] | Closed source | WD-based data | Cardiovascular measures, Blood Oxygen Saturation, Activity measures | NR |
| Kok [54] | Closed source | WD-based data | Acoustic | NR |
| Kueh [55] | Open source | WD-based data | Brain Activity | NR |
| Kusmakar [56] | Closed source | WD-based data | Activity measures | NR |
| Kusmakar [57] | Open source | WD-based data | Activity measures | 21 |
| Mehta [58] | Open source | WD-based data | Brain Activity | NR |
| Meisel [59] | Closed source | WD-based data | Electrodermal Activity, Skin temperature, Cardiovascular measures, Activity measures | NR |
| Milošević [60] | Closed source | WD-based data | Activity measures | 1680 |
| Milošević [61] | Closed source | WD-based data | Activity measures, Electromyography | NR |
| Mittlesteadt [62] | Open source | WD-based data | Cardiovascular measures | NR |
| Motahar [63] | Closed source | WD-based data | Activity measures | NR |
| Munch Nielsen [64] | Closed source | WD-based data | Brain Activity, Electrocardiography, and Activity measures | NR |
| Nasseri [65] | Closed source | WD-based data | Activity measures, Electrodermal Activity, Skin temperature, Cardiovascular measures | NR |
| Onorati [66] | Closed source | WD-based data | Activity measures, Electrodermal Activity | 65 |
| Prathaban [67] | Open source | WD-based data | Brain Activity | NR |
| Qian [68] | Open source | Non WD based data | Brain Activity | NR |
| R [69] | Closed source | Non WD based data | Activity measures | NR |
| Raj [70] | Closed source | WD-based data | Electrodermal Activity and Activity measures | NR |
| Regalia [71] | Closed source | WD-based data | Activity measures and Electrodermal Activity | NR |
| Seethalakshmi [72] | Closed source | WD-based data | Brain Activity | NR |
| Stirling [73] | Closed source | WD-based data | Cardiovascular measures, Sleep Stages, Step Count | 10 |
| Sutantos [74] | Closed source | WD-based data | Brain Activity | 24 |
| Tian [75] | Open source | WD-based data | Brain Activity, Electrocardiography, Electromyography | NR |
| Vandecasteele [76] | Open source | WD-based data | Brain Activity, Electrocardiography | 133 |
| Varun [77] | Open source | WD-based data | Brain Activity Electrocardiography | 2 |
| Vieluf [78] | Closed source | WD-based data | Electrodermal Activity, Skin temperature, Cardiovascular measures | 10 |
| Vieluf [79] | Closed source | WD-based data | Cardiovascular measures, Electrodermal Activity | NR |
| Wang [80] | Closed source | WD-based data | Activity measures, Electrodermal Activity, Electromyography, Skin temperature | 20 |
| Xianji [81] | Closed source | WD-based data | Activity measures | 21 |
| Yu [82] | Closed source | WD-based data | Activity measures, Cardiovascular measures, Electrodermal Activity | NR |
| Zhang [83] | Open source | WD-based data | Brain Activity | 21 |
| Zsom [84] | Closed source | WD-based data | Electrodermal Activity, Cardiovascular measures | 45 |
